# SARS-CoV-2 inhibitory activity of a short peptide derived from internal fusion peptide of S2 subunit of spike glycoprotein

**DOI:** 10.1016/j.virusres.2023.199170

**Published:** 2023-07-15

**Authors:** Maria Alfreda Stincarelli, Michael Quagliata, Andrea Di Santo, Lorenzo Pacini, Feliciana Real Fernandez, Rosaria Arvia, Silvia Rinaldi, Anna Maria Papini, Paolo Rovero, Simone Giannecchini

**Affiliations:** a Department of Experimental and Clinical Medicine, University of Florence, Viale Morgagni 48, Florence 50134, Italy; b Interdepartmental Research Unit of Peptide and Protein Chemistry and Biology, Department of Chemistry “Ugo Schiff”, University of Florence, Sesto Fiorentino 50019, Italy; c Interdepartmental Research Unit of Peptide and Protein Chemistry and Biology, Department of NeuroFarBa, University of Florence, Sesto Fiorentino 50019, Italy; d CNR - Istituto di Chimica dei Composti Organometallici (CNR-ICCOM), Via Madonna del Piano 10, Sesto Fiorentino I-50019, Italy

**Keywords:** Coronavirus, SARS-CoV-2, S2 Spike glycoprotein, internal fusion peptide region, synthetic peptide, antiviral activity

## Abstract

Severe acute respiratory syndrome coronavirus-2 (SARS-CoV-2) has posed a great concern in human population. To fight coronavirus emergence, we have dissected the conserved amino acid region of the internal fusion peptide in the S2 subunit of Spike glycoprotein of SARS-CoV-2 to design new inhibitory peptides. Among the 11 overlapping peptides (9-23-mer), PN19, a 19-mer peptide, exhibited a powerful inhibitory activity against different SARS-CoV-2 clinical isolate variants in absence of cytotoxicity. The PN19 inhibitory activity was found to be dependent on conservation of the central Phe and C-terminal Tyr residues in the peptide sequence. Circular dichroism spectra of the active peptide exhibited an alpha-helix propensity, confirmed by secondary structure prediction analysis. The PN19 inhibitory activity, exerted in the first step of virus infection, was reduced after peptide adsorption treatment with virus-cell substrate during fusion interaction. Additionally, PN19 inhibitory activity was reduced by adding S2 membrane-proximal region derived peptides. PN19 showed binding ability to the S2 membrane proximal region derived peptides, confirmed by molecular modelling, playing a role in the mechanism of action. Collectively, these results confirm that the internal fusion peptide region is a good candidate on which develop peptidomimetic anti SARS-CoV-2 antivirals.

## Highlights

• Peptides designed on internal fusion peptide region of S2 subunit of Spike SARS-CoV-2.
• High inhibitory activity of a short PN19 peptide from internal fusion peptide region.
• PN19 activity dependent to its α-helix structure.
• Key role of PN19 central Phe and C-terminal Tyr residues.
• Membrane proximal region derived peptide target of PN19 activity.

## 1 Introduction

The coronavirus associated disease 19 (COVID-19) pandemic highlighted that, in absence of an active vaccine, efficient antiviral drugs are needed (Zhu et al., 2020b; WHO 2023). In this context, the severe acute respiratory syndrome coronavirus-2 (SARS-CoV-2) cell entry has been used as an attractive target for monoclonal antibody therapy and to develop new antivirals (Mahendran et al., 2020; Agarwal and Gabrani 2021; Jackson et al., 2022). In particular, multiple interactions between the viral spike molecule and cellular substrates, including the S1 subunit interaction with cell surface angiotensin-converting enzyme-2 (ACE2) receptors and the S2 subunit mediating the final event of membrane fusion, represent a target of promising antivirals (Jackson et al., 2022; Marcink et al., 2022). However, SARS-CoV-2 variants challenged therapies using S1 subunit as a target of monoclonal antibodies and of peptides directed to the receptor binding domain (RBD) through its epitope changes on the viral spike glycoprotein (Jacobs et al., 2023). Thus, for its highly conserved features, so far, the S2 subunit has been the mainly used target to develop peptide inhibitors and to translate these compounds into therapeutic approaches (Shah et al., 2021; Huang et al., 2020). The S2 subunit is composed of fusion peptide (FP) and internal fusion peptide (IFP) regions, heptad repeat regions 1 and 2 (HR1-2), membrane proximal external region (MPER), transmembrane region (TM), and cytoplasmic tail (Jackson et al., 2022). For their molecular structures and role played during viral cell-fusion, HR1 and HR2 have been used as the targets of many studies based on peptides (Heydari et al., 2021; Tang et al., 2023; Mousavi et al., 2023). Mechanistically, peptides have been designed to mimic either HR1 or HR2 in the S2 subunit, in order to block formation of the HR1-HR2 6-helix bundle and thus interfering with the virus-host membrane fusion (Liu et al., 2004; Bosch et al., 2004). Among all peptides investigated until now, the pan-coronavirus fusion inhibitor EK1 and compound IPB-01, both 36-mer peptides designed on HR2 region, showed strong anti-CoV (SARS-CoV-1 and 2, and MERS-CoV) activity with IC_50_ values at micromolar concentrations (Xia et al., 2019a, 2019b,^2020^). Optimization of fusion inhibitors was also explored with rational modifications of the existing inhibitory peptides by linking a cholesterol group to C-terminus of EK1 (EK1C4) or of IPB01 (IPB02) (Xia et al., 2019a, 2019b,^2020^; Yu et al., 2021).

An additional point of interest in the development of antiviral peptides is the use of the FP, IFP and MPER region in the S2 subunit of SARS-CoV-2 as potential target. To date, although the FP insertion in the host membrane and the subsequent formation of HR1 and HR2 interactions to mediate the viral cell-fusion is a well understood process, the subsequent events and the role of IFP and MPER and their contribution to membrane fusion remain to be elucidated in detail (Liao et al., 2015). MPER functional role in SARS-CoV infection and fusion activity was investigated identifying residues playing a major role (Cover et al., 2009). Moreover, it has been reported that MPER sequence coupled to HR2 derived peptide increase the antiviral activity (Zhu et al., 2020a, 2022; Yu et al., 2021). In the context of these molecular interactions, it is of relevance the common hydrophobic features of MPER and IFP region, the presence of conserved aromatic amino acid residues (Trp, Phe, Tyr), and the fact that mutation analysis of these residues confirmed the impairment of viral cell fusion activity (Liao et al., 2015; Cover et al., 2009). Of note, all coronavirus spike glycoproteins share a highly conserved MPER and IFP (Ou et al., 2016).

Prompted by all these information, we considered of interest to explore the potential inhibitory activity of peptides designed on the basis of SARS-CoV-2 S2 IFP region. To do this, we initially considered peptide P23, belonging to the IFP region of the S2 subunit of SARS-CoV-2 and corresponding to the previously reported peptide IFP23, which was shown to interact with the MPER of SARS-CoV (Liao et al., 2015). The inhibitory activity against SARS-CoV-2 variants of P23 and its derivatives with a reduced size lead us to identify a short peptide analogue, PN19, exerting high inhibitory activity. Experiments to evaluate the PN19 structure-activity relationship, as well as the mechanism of action and the potential target of its inhibitory activity were also carried out.

## 2 Materials and methods

### 2.1 Cells and viruses

The cell lines used were Vero E6 (CRL-1586, ATCC, Rockville, Md, USA) cultivated using Dulbecco's Modified Eagle's Medium (DMEM) supplemented with 10% fetal bovine serum (FBS). In some experiments Vero E6 cells expressing Transmembrane serine protease 2 (TMPRSS2) were also used. The viruses used were SARS-CoV-2 clinical isolates (SCV2/Fi/3/22 Wuhan-like with the mutation G614D pango lineage B.1, SCV2/Fi/1/21 Alpha pango lineage B.1.1.7, SCV2/Fi/2/21 Delta pango lineage B.1.617.2, and SCV2/Fi/1/22 omicron pango lineage BA.1; spike glycoprotein mutation reported in Tables 2s) grown on Vero E6 cells, titrated by the plaque method, aliquoted and stored at -80°C until used.

### 2.2 Peptide synthesis and purification

Peptides were synthesized by Magnetic Induction-assisted Solid-Phase Synthesis following the Fmoc/tBu strategy, using the PurePep®Chorus® automated peptide synthesizer (Gyros Protein Technologies, Uppsala, Sweden). Synthesis protocols and full analytical data (HPLC and MS) are reported in the Supplementary Material (Figs. 1s-25s).

**Fig. 1:**
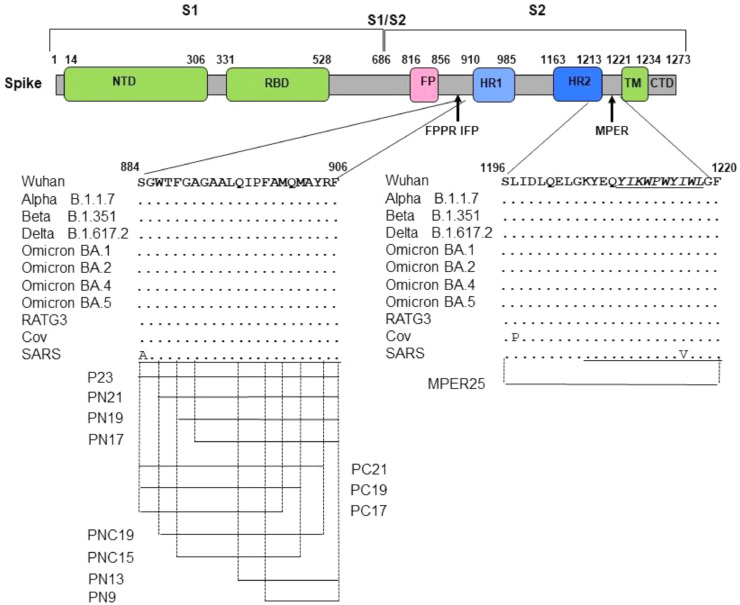
Schematic representation of the Spike glycoprotein S1/S2 subunit of SARS-CoV-2 and the localization of peptides used in the study. Variability of the peptide sequence in the S2 subunit for SARS-CoV-2, including SARS-CoV-2 variants (Table 2s) used in this study pango lineage B.1 (Wuhan-like with the mutation G614D), B.1.1.7 (Alpha), B.1.617.2 (Delta), BA.1 (Omicron), SARS-CoV, bat SARS-like CoV and Pangolin CoV are also showed. Underline sequence represent region of peptide used in other study (Liao et al., 2015). NTD, N-terminal domain, RBD, receptor binding domain, FP, fusion peptide, FPPR, Fusion peptide proximal region, IFP, internal fusion peptide, HR1, heptad repeat 1, HR2, heptad repeat 2, MPER, membrane proximal region, TM, transmembrane region, Cyto, cytoplasmic region.

### 2.3 Circular dichroism

CD spectra of the peptides were recorded using quartz cells of 0.1 cm path length with a JASCO J-810 CD spectropolarimeter at 25°C, in the 260−190 nm spectral range, 1 nm bandwidth, 4 accumulations, and 50 nm/min scanning speed. The peptides were dissolved in H_2_O:TFE 1:1 (v/v) at a concentration of 200 μM. The secondary structure content of the peptides was predicted using the online server for protein secondary structure analyses BestSel (Miesonai et al., 2018).

### 2.4 Structural prediction and molecular modeling

*Structural prediction.* Secondary structure prediction of peptides was performed using PEP-FOLD server (https://bioserv.rpbs.univ-paris-diderot.fr/services/PEP-FOLD, accessed on 10 March 2023;Shen et al., 2014; Thèvenet et al., 2012; Pace and Sholtz, 1998).

*Molecular modelling*. Due to the lack of structural information about the membrane proximal external region of SARS-CoV-2 spike protein, predicted 3D models of the trimer resulting from an optimized protocol described previously (Izvorski, 2020) were used for modelling the interaction with PN19 peptide and its Ala-mutated analogs. In order to increase the significance of the binding predictions, we used the most representative models (for a total of five). NMR structures of the target peptide (PDB ID: 2ruo) (Mahajan and Bhattacharjya 2015), were obtained from Protein Data Bank (http://www.rcsb.org). Maestro Program (Schrödinger, 2021) was used to process the peptide structure and to predict the Phe888, Gly889 and Ala890 missing residues. The structures were finally subjected to OPLS3 force field (Harder et al., 2016) energy optimization. HpepDock protocol (Zhou et al., 2018) was used as gold standard software for flexible peptide-protein docking (Weng et al., 2020) with standard parameters. Solvent-exposed residues of each MPER monomer were indicated as putative binding site (1203-LGKYEQYIKWPWY-1215). By means of Robetta Server, (Kortemme and Baker, 2022) the complex with the highest docking score for each of the 5 MPER models was used for *in silico* Alanine Scanning mutagenesis. Structures were visualized and analyzed with VMD software (Humphrey et al., 1996).

### 2.5 Inhibition of SARS-CoV-2 infection

*Inhibitory experiment.* Six-well plate was seeded with 2.5 × 10^5^ Vero E6 cells in 3 mL of growth medium and kept overnight at 37°C with 5% CO2. Unless otherwise stated, SARS-CoV-2 viral stock to produce a final multiplicity of infection (MOI) of 0.01 was mixed 1:1 in a final volume of 0.3 mL with 10-fold serial dilutions (final concentrations of 100, 10, 1, 0.1 μM) of peptides (resuspended in DMSO), and immediately added onto the cell-substrate and incubated for 1 h at 37°C with 5% CO2. Virus mixed with DMSO alone was used as control. Then, cells were washed with PBS 1X and the overlay medium composed by 0.5% Sea Plaque Agarose diluted in propagation medium in absence of FBS was added to each well. After 3 days of incubation at 37°C, the monolayers were fixed with methanol, stained with 0.1% crystal violet and the viral titers was calculated by plaque-forming-unit (PFU) counting. Percent of Plaque reduction activity was calculated by dividing the average of PFU of treated samples by the average of DMSO-treated samples (viral positive control). Fifty percent inhibitory concentrations (IC_50_) was calculated using the predicted exponential growth function in Microsoft Excel.

*Time of addition experiment.* The time of action of selected peptide was investigated performing the experiment of inhibitory activity as described previously in presence of peptide at 0.1, 1, 10 and 100 μM concentration, added 1 h before infection (T-1), at the time of infection (T0) and 1 h (T1) and 2 h (T2) post infection.

*Peptide adsorption experiment.* For adsorption experiment, first the SARS-CoV-2 was incubated with Vero E6 cells (5 × 10^5^) at 4°C (to permit virus adsorption but not entry) or at 37°C (to permit both virus adsorption and entry) for 1 h. Then, after removal of virus inoculum, peptide (1 mM solution) was incubated with the SARS-CoV-2 adsorbed to Vero E6 cells or with Vero E6 cells alone at 4°C for 1 h, centrifuged at 600 X g for 15 min (to clarify the residual peptide solution) followed by 20,000 X g for 90 min (to remove residual virions). The residual inhibitory activity in the peptide solution obtained after adsorption treatment was assayed for inhibition of the same SARS-CoV-2 used in peptide adsorption treatment, by the standard procedure. The peptide solution after such treatment inoculated alone in the Vero E6 cells exhibited absence of virus infectivity.

*Peptide mixing experiment.* To investigate the potential effect of peptide from MPER region on active peptide, selected peptide was used alone or in presence of peptide MPER25 diluted to obtain single final concentrations from 0.05 to 50 μM, incubated at 4°C for 30 min, and then assayed for SARS-CoV-2 inhibition by the standard procedure.

All experimental procedures were performed under biosafety level-3 (BSL3) containment.

### 2.6 Cell cytotoxicity assay

Vero E6 cells were plated at a density of 10^4^ cells per well in a flat-bottom 96-well culture plate and allowed to adhere overnight. When the cell layers were confluent, the medium was removed, the wells were washed twice with PBS and treated with 100 μl of DMEM alone or with the appropriate concentrations of the peptides (the final concentration added to the cells ranged from 0.1 to 100 μM) and incubated at 37°C in a CO_2_ incubator for 72 h. After treatment, an MTT kit (Roche, Milan, Italy) was used according to the supplier's instructions. Cytotoxicity was calculated by dividing the average optical density of treated samples by the average of the mock-treated samples in the presence of DMSO alone.

### 2.7 Surface plasmon resonance (SPR) experiments

Experiments were performed using the Biacore® X100 (Cytiva, Milano, Italy). All binding analyses were made at 25°C using HBS-EP+ as running buffer. All the experiments were conducted as previously described (Real Fernandez et al., 2015; Cimitan et al., 2005), after proper optimization for the present study (supplementary information).

### 2.8 Statistical analysis

Data were analyzed using a two-tailed *Student's t-test*. All data represent three independent experiments with p<0.05 considered statistically significant.

## 3 Results

### 3.1 SARS-CoV-2 inhibitory activity of peptides derived from S2 internal fusion peptide region

**Fig. 2:**
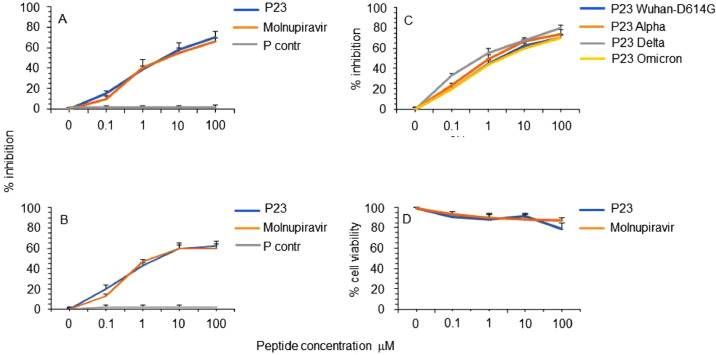
Inhibitory activity and cytotoxicity of peptide PN23 against SARS-CoV-2 Vero E6 cell infection. SARS-CoV-2 infection of Vero E6 cells at MOI of 0.01 (A) and of 0.1 (B) in presence of the indicated concentration of PN23 peptide and molnupiravir reference inhibitor was assayed with the viral plaque reduction assay. Peptide control (P contr) with the same length but with unrelated sequence was also used. (C) SARS-CoV-2 (Wuhan-G614D, SCV2/Fi/3/22 Wuhan-like with the mutation G614D pango lineage B.1; Alpha, SCV2/Fi/1/21 Alpha-like pango lineage B.1.1.7; Delta, SCV2/Fi/2/21 Delta-like pango lineage B.1.617.2; Omicron, SCV2/Fi/1/22 omicron-like pango lineage BA.1) infection of Vero E6 cells at MOI of 0.01 in presence of the indicated concentration of P23 peptide was assayed with the viral plaque reduction assay. (D) Cell viability of Vero E6 cells in the same condition in absence of virus infection was assessed with MTT assay. The values shown are means ± standard deviation (SD) of 3 independent experiments.

We initially assayed the inhibitory activity of peptide P23, derived from IFP region in the S2 of SARS-CoV-2 and corresponding to the homolog amino acid region of peptide IFP23 of SARS-CoV, proved to play a role in the fusion mechanism (Liao et al., 2015). The selected region is highly conserved among the SARS-CoV, SARS-CoV-2 main variants emerged till now and also with other non-human coronavirus (Fig. 1). Fig. 2A,B shows that P23 exhibited a powerful dose dependent inhibitory activity of different SARS-CoV-2 virus infectious doses (IC_50_ mean ± SD of 2.7±1.1 and 5.3±2.3 μM at MOI 0.01 and 0.1, respectively). Of note, the activity of P23 was similar to that of the inhibitor molnupiravir, a nucleoside analogue acting via lethal mutagenesis on viral RNA and active against SARS-CoV-2, taken as positive control of inhibition. An unrelated peptide of the same length, used in the same condition, was not active. Moreover, the peptide P23 was active against different SARS-CoV-2 variants used (Mean of IC_50_ ranged from 0.8 to 3.4 μM, Fig. 2C). Importantly, under the experimental conditions used, P23 did not exert significant reduction in cell viability (Fig. 2D).

### 3.2 SARS-CoV-2 inhibition by peptides of reduced size

Then, to dissect the minimal sequence involved in the P23 antiviral activity reported in Fig. 2, we synthetized peptides with reduced length, as reported in Fig. 1. In particular, the short peptides were generated reducing P23 by 2-4 residues in the N-terminal or C-terminal (PN derivatives and PC derivatives, respectively) or in both termini (PNC derivatives). Table 1 shows the inhibitory activity of the 10 short peptides against SARS-CoV-2 infection in Vero E6 cells, featuring an IC_50_ ranging from 0.20 to 97.94 μM. Of note, among the shorter peptide endowed with significant activity, the 19-mer peptide PN19 (P23 reduced by 4 residues in the N-terminal) was the more active, while peptide PC17 (P23 reduced by 6 residues in C-terminal) the less active. Peptide PNC15 (P23 reduced by 4 residues in both N- and C-termini) lost completely inhibitory activity. Of note, examining all the shorter peptides it emerged that the major effect in the impairment of the inhibitory activity was obtained with the deletion by 1-6 residues in the P23 C-terminal., as shown by the maintained activity exerted by PN9 and PN13 (Table 1 and Figure 26s). All peptide derivatives did not exert significant reduction in cell viability. Finally, PN19 peptide, assayed against SARS-CoV-2 infection in Vero E6 cells expressing TMPRSS2, maintain its effectiveness demonstrating that inhibitory activity was not affected by the presence of TMPRSS2 (Table 1).

**Table 1:** Inhibitory activity of reduced size peptide P23

Peptide	Sequence	IC_50_ (mean ± SD) μM	CC_50_ (mean ± SD) μM
Vero E6 cell substrate			
P23	^884^SGWTFGAGAALQIPFAMQMAYRF^906^	2.80 ± 2.68	> 100
PN21	^886^WTFGAGAALQIPFAMQMAYRF^906^	0.71 ±2.11	> 100
PN19	^888^FGAGAALQIPFAMQMAYRF^906^	0.20 ±1.11	> 100
PN17	^890^AGAALQIPFAMQMAYRF^906^	2.79 ±1.96	> 100
PC21	^884^SGWTFGAGAALQIPFAMQMAY^904^	8.33 ±3.60	>100
PC19	^884^SGWTFGAGAALQIPFAMQM^902^	9.43 ±2.44	> 100
PC17	^884^SGWTFGAGAALQIPFAM^900^	97.94 ±21.84	> 100
PNC19	^886^WTFGAGAALQIPFAMQMAY^904^	46.83 ±9.97	> 100
PNC15	^888^FGAGAALQIPFAMQM^902^	>100	> 100
PN13	^894^LQIPFAMQMAYRF^906^	1.70 ±0.52	> 100
PN9	^898^FAMQMAYRF^906^	5.93 ±9.74	> 100
Vero E6/TMPRSS2			
PN19	^888^FGAGAALQIPFAMQMAYRF^906^	1.10 ± 0.11	> 100

^a^Values obtained in two to three independent assays.

### 3.3 Importance of the Phe and Tyr residues for inhibitory activity of peptides

The result obtained in the experiments of size reduction of peptide P23 indicated that PN19 is the most active. Of interest, this 19-mer peptide contains 3 Phe residues (Phe at both termini and Phe in the central region) separated by 9 and 7 residues in the sequence, respectively. To assess the importance of each single residue for inhibitory activity of peptide PN19, it was performed an Ala scan study, in which PN19 modified peptides were compared for anti-SARS-CoV-2 activity with the wild-type peptide, thus obtaining information about the role played by each side chain. As shown in Table 2 (figure 27s), Ala substitution in PN19 peptide for Phe-898 and Tyr-904 markedly reduced antiviral effectiveness. All other Ala substitutions had little (PN19 substitution in Phe-888, Glu-895 and Glu-901, Phe-906) or no effect. Thus, these findings suggest that the presence of a central Phe residue and a C-terminal Tyr residue are crucial for robust inhibitory activity.

**Table 2:** Effect of alanine scan on SARS-CoV-2 inhibitory activity of peptide PN19

Peptide	Sequence	IC_50_ (mean ± SD) μM	CC_50_ (mean ± SD) μM
PN19_wt_	^888^FGAGAALQIPFAMQMAYRF^906^	0.80 ± 1.68	> 100
PN19_F→A888_	^888^AGAGAALQIPFAMQMAYRF^906^	2.91 ± 1.95	> 100
PN19_L→A894_	^888^FGAGAAAQIPFAMQMAYRF^906^	0.60 ± 0.42	> 100
PN19_Q→A895_	^888^FGAGAALAIPFAMQMAYRF^906^	3.82 ± 3.50	>100
PN19_I→A896_	^888^FGAGAALQAPFAMQMAYRF^906^	0.30 ± 1.40	> 100
PN19_P→A897_	^888^FGAGAALQIAFAMQMAYRF^906^	0.24 ± 0.14	> 100
PN19_F→A898_	^888^FGAGAALQIPAAMQMAYRF^906^	57.73 ± 66.17	> 100
PN19_M→A900_	^888^FGAGAALQIPFAAQMAYRF^906^	0.84 ± 0.52	> 100
PN19_Q→A901_	^888^FGAGAALQIPFAMAMAYRF^906^	3.10 ± 0.82	> 100
PN19_M→A902_	^888^FGAGAALQIPFAMQAAYRF^906^	1.41 ± 2.42	> 100
PN19_Y→A904_	^888^FGAGAALQIPFAMQMAARF^906^	28.32 ± 47.96	> 100
PN19_R→A905_	^888^FGAGAALQIPFAMQMAAAF^906^	0.85 ± 0.72	> 100
PN19_F→A906_	^888^FGAGAALQIPFAMQMAYRA^906^	2.65 ± 2.45	> 100

^a^Ala scan was performed in all amino acid residues of PN19 except for Ala and Gly residues of wild type sequence. Values obtained in two to three independent assays.

### 3.4 CD spectra and secondary structure prediction

**Fig. 3:**
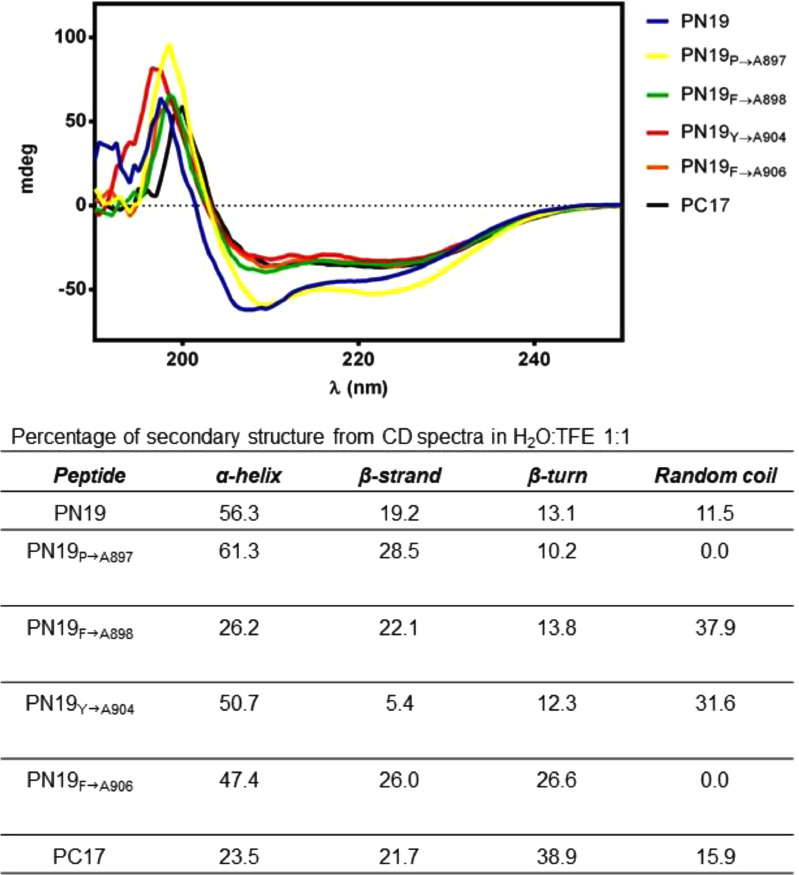
CD spectra of selected peptides in H_2_O:TFE 1:1. CD spectra was performed for PN19 and PN19_P→A897_ as most active peptides, and for PN19_F→A898,_ PN19_Y→A904_,PN19_F→A906_ and PC17 to test the role of central Phe, Tyr and terminal Phe respectively. Mdeg, millidegrees.

**Fig. 4:**
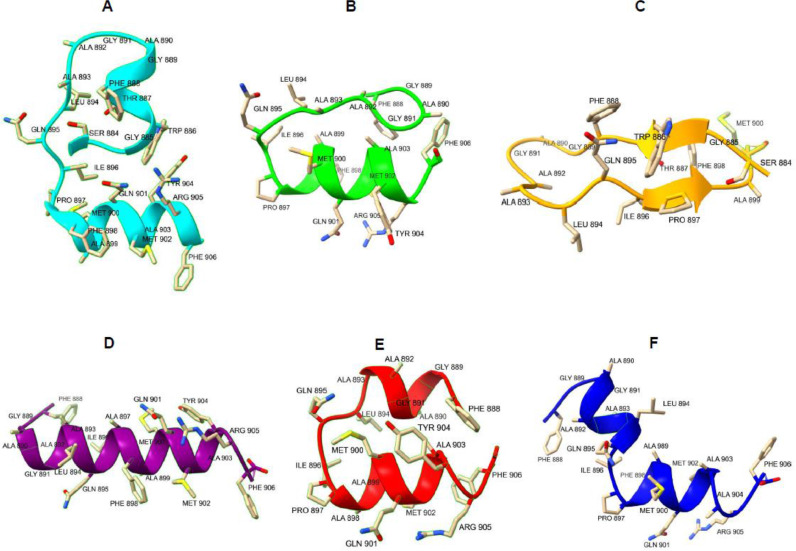
Secondary structure predictions of (A) native P23 peptide; (B) PN19 peptide and (C) PC17 peptide; (D) PN19_P→A897_; (E) PN19_F→A898_ and (F) PN19_Y→A904_.

The secondary structure propensity of some peptides was studied by CD spectroscopy (Fig. 3). In particular, we selected PN19 and PN19_P→A897_, as the most active peptides, and PN19_F→A898_, PN19_Y→A904_, PN19_F→A906_, to test the role of central Phe, and C-terminal Tyr and Phe, respectively. Also, we selected PC17 as an inactive peptide. In general, all the sequences tend to assume an α-helical structure, except for PN19_F→A898_, and PC17, which are also the least active. On the other hand, PN19 and PN19_P→A897_ have the most ordered structure, showing two deep minima at 209 nm and 222 nm. In light of these considerations, the α-helical structure appears to be essential for the antiviral activity of the peptides, and the central Phe residue plays a key role in both structure and activity. At variance, Tyr and terminal Phe residues seem to have less influence on the stability of the secondary structure (Fig. 3). The secondary structure predictions obtained using the PEP-FOLD server indicate that both PN19 and P23 show a significant presence for an α-helical conformation between residues Pro897 and Arg905 (Fig. 4A,B). In contrast, the simulated structure of the fragment PC17 shows the presence for a β-sheet (Fig. 4C), which is not present in the native peptide structure. In order to observe if there were differences changing some key residues, considering the sequence of peptide PN19_P→A897_, it is noted that the Pro897Ala mutation stabilizes the α-helix, as alanine has much more helix propensity than proline (3.16 vs 0.00 kcal/mol; Fig. 4D). On the other hand, the sequence of PN19_F→A898_ and PN19_Y→A904_ appear to maintain an α-helix only in the C-terminal portion. Such result suggests that the reduced activity of the mutated peptides Phe898Ala and Tyr904Ala, compared to not mutated PN19, may be related to the altered conformation of these peptides, which affect the key role played by the two aromatic residues (Fig. 4E,F).

### 3.5 Effect the time-addition and use of MPER target on Peptide PN19 inhibitory activity

**Fig. 5:**
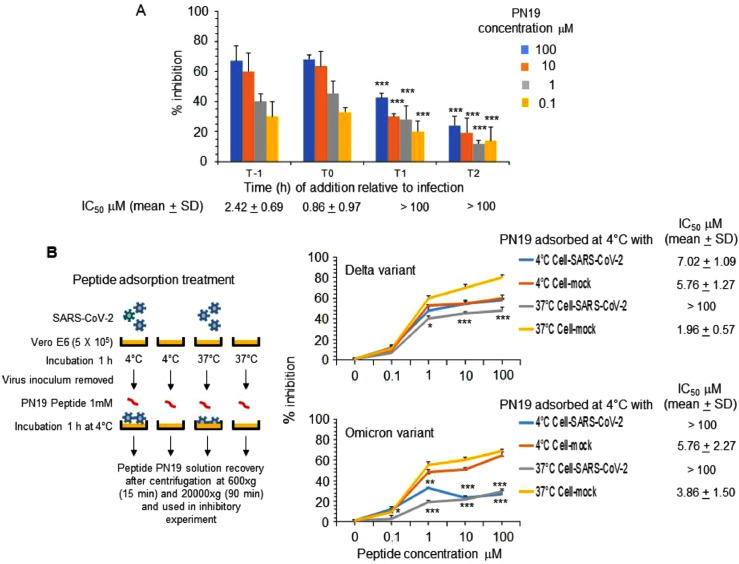
Effect of varying peptide PN19 time of administration and adsorption with SARS-CoV-2 cell mixtures and substrate cells on SARS-CoV-2 replication. A- SARS-CoV-2 infection of Vero E6 cells at MOI of 0.01 in presence of peptide PN19 at 0.1, 1, 10 and 100 μM concentration added at 1 h before infection (T-1), at the time of infection (T0) and 1 h (T1) and 2 h (T2) after infection. SARS-CoV-2 infection exposed to the indicated doses of PN19 was assayed with the viral plaque reduction assay. B- In the adsorption treatment Peptide PN19 (1 mM solution) was incubated at 4°C for 1 h with the indicated material: SARS-CoV-2 adsorbed to Vero E6 cells (5 × 10^5^) (Cell-SARS-CoV-2) or Vero E6 cells (5 × 10^5^) (Cell-mock) alone for 1 h at 4°C or at 37°C. Then, peptide solution after adsorption treatment was centrifuged at 600 X g for 15 min (to remove cells) followed by 20,000 X g for 90 min (to remove residual virions), and then assayed at the concentration indicted for inhibition of the same SARS-CoV-2 used in PN19 adsorption treatment, by the standard procedure. The peptide PN19 solution after such treatment inoculated alone in the Vero E6 cells exhibited absence of virus infectivity. Values shown are means ± standard deviations (SD) of the inhibitory values obtained in 3 independent experiments. Asterisks indicate significative difference at * p<0.05, ** p<0.01, *** p<0.001 *(Student's t-test).*

**Fig. 6:**
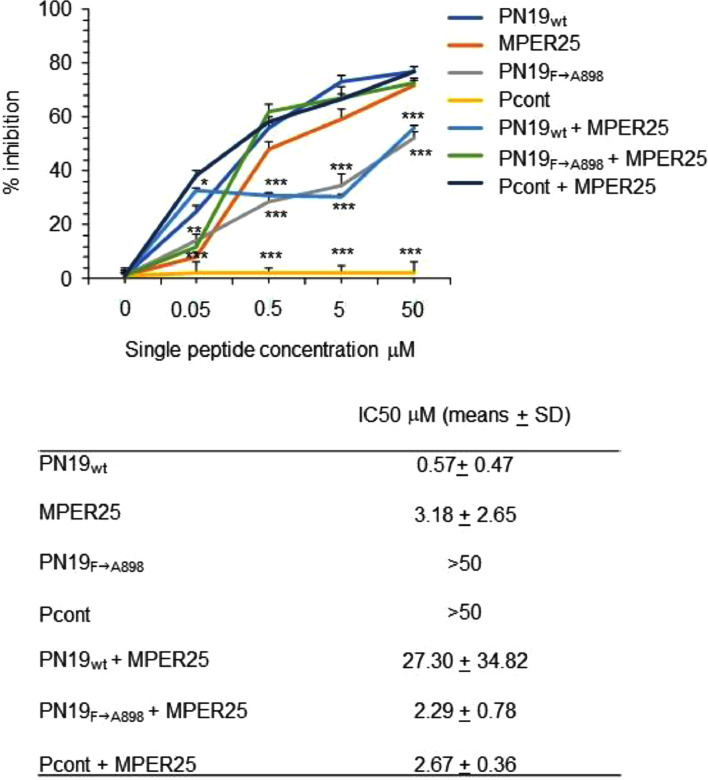
Effect of mixing PN19 peptides with MPER25 peptide on inhibitory activity of peptide PN19 against SARS-CoV-2 in Vero E6 cells infection. Peptide PN19 wilt type, PN19_F→A898_ and MPER25 alone diluted to contain single final concentrations from 0.05 to 50 μM alone or mixed were incubated at 4°C for 30 min, and then assayed for SARS-CoV-2 inhibition by the standard procedure. Peptide control with the same length but with unrelated sequence was also used. The values shown are means ± standard deviation (SD) of 3 independent experiments. Asterisks indicate significative difference at * p<0.05, ** p<0.01, *** p<0.001 *(Student's t-test).*

**Fig. 7:**
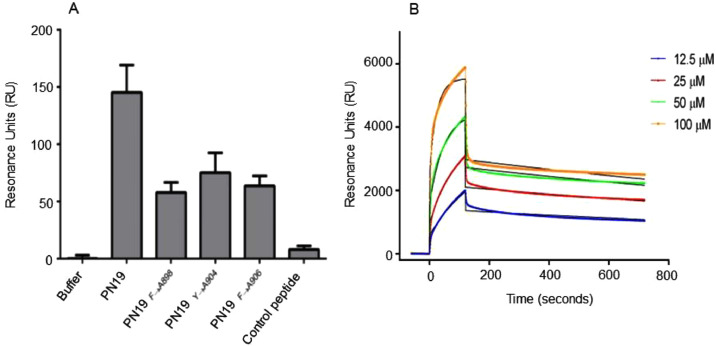
MPER25c SPR binding activity of PN19 peptide and its derivatives. A- Binding levels to immobilized MPER25c peptide of peptide PN19 compared to PN19_F→A898_, PN19_Y→A904_, PN19_F→A906_ at the concentration 100 µM. peptide MPER25c was covalently immobilized onto the gold surface of the biosensor following the thiol coupling immobilization strategy. All peptides were flowed separately over the immobilized MPER25c fragment in individual cycle of analysis with an initial association phase (sample injection), a second dissociation phase (washing step with running buffer), and finally a chip surface regeneration phase. Background levels due to non-specific peptide interactions were monitored on the reference channel of the chip and subtracted from sample signals. The running buffer and a control peptide has been also tested as negative controls. B- Sensorgrams of interaction between peptide PN19 and the immobilized MPER25c at different concentrations comprising the theoretical curves for a 1:1 binding model (black).

**Fig. 8:**
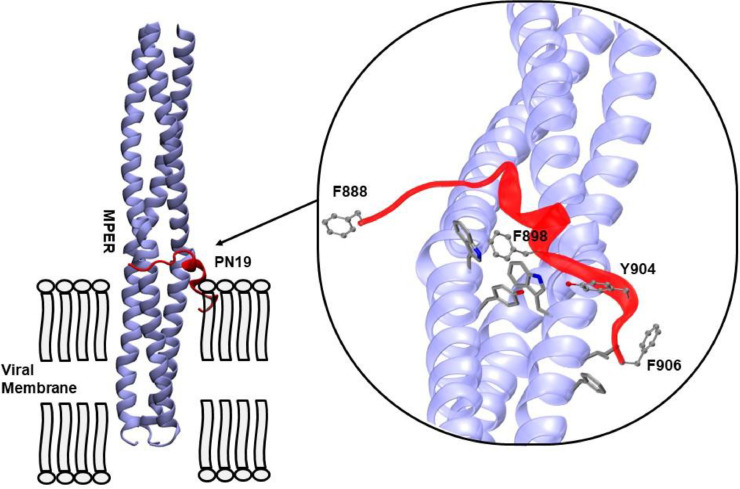
Molecular modelling of PN19 interaction with the MPER spike region. Representative binding pose conformation resulted from molecular docking analysis using the optimized Spike structure reproducing sequence from 1172-1240 described previously (Izvorski, 2020). Viral membrane is reported. Zoom on the region of interaction between the MPER region (Blue) and the PN19 peptide (Red). Aromatic amino acids of PN19 peptide (Phe888, Phe898, Tyr904, and Phe906) are shown in CPK visualization mode of VMD. The MPER target amino acids (Tyr, Phe, Trp and Ile) within 3 Å from the PN19 hotspots are represented in licorice.

Time of addition studies, performed at different PN19 concentrations, clearly demonstrated that PN19 acted targeting a step in the early time (1 h) of infection of the SARS-CoV-2 life cycle (Fig. 5A). Accordingly, it was considered of interest to explore if the peptide activity was mediated by its adsorption to the cells substrate alone or after virus binding and cell entry. Thus, to investigate the possible target of PN19 activity, peptide PN19 was incubated at 4°C with either Vero E6 cells alone, or Vero E6 cells adsorbed with SARS-CoV-2 in condition that permitted virus-cell binding and virus cell-entry (Fig. 5B). Then, peptide residual inhibitory activity in the supernatant after adsorption treatment was determined against SARS-CoV-2. Fig. 5B showed that adsorption treatment of PN19 with SARS-CoV-2 Delta variant virus-cell mixture pre-incubated under conditions which permitted virus adsorption to cells and entry, but not under conditions which permitted cell adsorption and not entry, exhibited the capacity to remove peptide PN19 activity. On the other hand, adsorption of peptide with SARS-CoV-2 omicron variant removed much of the peptide PN19 activity, even in the condition that did not permit cell entry. Of note, adsorption with Vero E6 cells alone had a negligible effect on the inhibitory properties of peptide PN19. Thus, these findings showed that SARS-CoV-2 capacity to bind PN19 depends on the nature of spike and occur mainly after virus has adsorbed and entry onto cells. Then, we examined whether PN19 activity was affected by mixing with peptides derived from the C-terminal portion of Spike glycoprotein (MPER25 peptide from MPER region, Fig. 1). As shown in Fig. 6, peptide MPER25 was active against SARS-CoV-2 (Mean±SD of IC_50_ of 3.18±2.65 μM). Of note, peptide MPER25 produced a 48-fold reduction of the expected IC_50_ value of PN19. The antiviral effect in control cultures in which PN19 was substituted with PN19_F→A898_ showed a lower reduction, compared to PN19 (mean of IC_50_ of 2.29 versus 0.57, for PN19 and PN19_F→A898_, respectively). At variance, the antiviral activity of MPER25 peptides was unaffected by the presence of a control peptide. Finally, to investigate the molecular interaction of peptide MPER25c (peptide MPER25 with Ser substituted with Cys for technical purpose) with PN19 and its mutated analogues, we performed binding experiments, using SPR. Fig. 7A shows that PN19 binds to immobilized MPER25c, while the Ala-mutated analogues PN19_F→A898_, PN19_Y→A904_, and PN19_F→A906_ exhibited at least 2-3-fold lower binding activity. Then, we performed a kinetic study testing different concentrations of peptide PN19 on the immobilized MPER25c peptide. Binding results were elaborated independently for each sample concentration, fitting the experimental values to the most suitable theoretical kinetic models. Thus, the affinity constant KD = 9.44 nM (kd = 3.88 × 10-4 s-1/ka = 410.9 M-1 s-1) were calculated describing the interaction between PN19 and MPER25c peptide. Finally, molecular docking was used to validate the proposed mechanism and unveil the atomistic details of the interaction between PN19 and MPER S2 subunit. The C3 symmetry of the trimeric spike protein and the conformational flexibility of the MPER region (Izvorski,2020) result in a variability of calculated docking poses (supplementary figure 28s). It is interesting to note, however, that the most recurring binding conformations optimize the number of interactions with all three monomers of the spike protein resulting in docking poses characterized by horizontal orientation along the trimer axis and in close proximity to cell membrane. Furthermore, the binding has a minimal impact of the peptide's secondary structure in accordance with the CD data. *In silico* alanine scanning performed on the most probable binding poses (see Methods) confirm that the aromatic residues of PN19 act as stabilization hotspots for the peptide-target complex (Fig. 8 and Table 3s). Specifically, Phe898 engages in a network of hydrophobic interactions with the target's aromatic amino acids, whose perturbation would explain both the impact on the secondary structure stability and the reduced activity of the Phe898Ala mutant.

## 4 Discussion

In this study, among several peptides (9-23 mer) derived from IFP region of the S2 subunit of SARS-CoV-2 spike glycoprotein, PN19 (19-mer) exerted high inhibitory activity mapped in a short segment containing three Phe residues, separated from one another by eight and nine amino acids, respectively. Of note, PN19 exhibits a high propensity for α−helix structure and its activity markedly dependent on the presence of a central Phe and a C-terminal Tyr residue. PN19 peptide exerted inhibitory activity in the first step of virus infection, and its activity was removed mainly by incubation treatment with SARS-CoV-2 variants adsorbed onto substrate cells in a condition allowing fusion, but not by-incubation treatment with the same virus-cell adsorbed in a condition which had not permitted virus entry, or with cell substrate alone. In this regard, the ability of omicron variant to adsorb the peptide activity also in virus cell condition that not permitted virus entry likely reflected the difference in the stability of Spike glycoprotein and fusion ability exerted by this variant (Suzuki et al., 2022). Finally, the inhibitory activity of PN19 was found to be blocked by peptide MPER25, corresponding to a conserved segment contained in the MPER motif at the C-terminus of S2 subunit of SARS-CoV-2. Binding measurements confirms that PN19 was able to specifically interact with MPER25 peptide but reduced its binding if central Phe898, and C-terminal Tyr904 and Phe906 residues were substituted with Ala.

Molecular modelling prediction of interaction analysis confirmed that the PN19 binding activity was mediated principally by the role of Phe898. Collectively these results point out that PN19 inhibitory activity depends upon both structural aspects, and on the specific role played by the central Phe898 and the C-terminal Tyr904 residues, as critical determinants for the binding stability with the target region. In this regard, the reduced binding of the peptide derivative mutated at Phe898 correlated to a reduced α-helix stability, as compared to that of PN19. However, the reduced binding of this mutated peptide and of that mutated at Tyr904 likely depends also from other features, involving specific side chain binding interactions. In particular, since the MPER motif binding activity of these mutated peptides correlates poorly with the inhibitory activity, additional specific interactions of Phe898 and Tyr904 residues to different target(s) may be hypothesized. The reduced binding activity of peptide mutated at Phe906, which on the contrary maintain inhibitory activity comparable to that of the not mutated peptide, is in line with this latter hypothesis. Thus, in the inhibitory activity of these mutated peptides may potentially play a role the interaction to the MPER target in its trimeric form and in the context of membrane environment at the end of viral cell-fusion. All together, these structure-activity relationship data confirm the role of Phe and other aromatic residues in the reproduction of an α-helix stable structure for the viral fusion activity of the IFP, as reported previously for SARS-CoV (Ou et al., 2016; Mahajan and Bhattacharjya, 2015). In this context, the role of membrane in stabilizing the functional feature of peptides is a matter of debate, as reported in molecular investigation of SARS-CoV-2 IFP structure-activity relationship (Mahajan and Bhattacharjya, 2015; Pattnaik et al., 2021; Yang et al., 2022; Ling et al., 2020; Sardar et al 2023).

To date the study of the structure-activity relationship for SARS-CoV-2 S2 peptides derived from the HR2 region demonstrated that an α-helix structure plays a dominant role to block formation of the HR1-HR2 6-helix bundle thus interfering with the virus-host membrane fusion (Heydari et al., 2021; Tang et al., 2023). The localization of our PN19 peptide and its ability to bind MPER derived peptide could indicate a different mechanism of inhibitory activity. Molecular evidence showed that juxtaposition of the S2 IFP and MPER domains facilitates the formation of the fusion pore through lipid destabilization in the late steps of viral cell membrane fusion (Behzadipour and Hemmati, 2022; Palombi et al., 2021; Santamaria et al., 2022; Mahajan and Bhattacharjya, 2015). Thus, the interaction between the IFP and the MPER in membrane context would be one of the most important targets where peptide PN19 exerted its mechanism of action. Proving unequivocally the latter aspect will, however, require a finer dissection of the phenomenon.

Development of entry and fusion powerful inhibitory peptide have recently increased the clinical interest for their use in treatment of SARS-CoV-2 infection (Heydari et al., 2021). In this context, new small compounds targeting membrane envelope viral structure, as inhibitors of viral entry, can be of value (Tang et al., 2023). A peculiar aspect of the present study is the small size of some S2 SARS-CoV-2 inhibitory peptides studied. As a rule, oligopeptides of such small size are devoid of consistent activities due to absent or poor conformational stability. Indeed, all consistently effective SARS-CoV-2 fusion-inhibitory peptides described to date are composed of 26 or more residues and additionally sometime contain membrane interacting moieties, such as cholesterol (Pattnaik et al., 2021; Yang et al., 2022; Ling et al., 2020; Liu et al., 2009). Likely, the presence in PN19 of three equally distanced Phe residues may determine a folded compact structure which appeared to correlate with inhibitory activity, since substitution of Ala for Phe989 greatly affected CD features and reduce or eliminated inhibitory activity. Moreover, this reduced size is important to also reduce the potential immunogenicity of the peptide, that is relevant in the development of peptide mimetic compound for possible use in vivo. The presence of the aromatic side chains of Phe and Tyr in the peptide, is also suggestive of a possible direct involvement of these residues in hydrophobic interaction with the PN19 target site. Noteworthy aromatic residues in the IFP region of S2 SARS-CoV-2, as well as in other coronavirus, are highly conserved (Ou et al., 2016) and, as determined by mutagenesis and crystal structure analyses, the target cavity in which SARS-CoV-2 IFP and MPER are believed to exert their action are susceptible to be filled by small-molecule compounds. At the same time, the high sequence conservation of both IFP and its target suggests that the occurrence of escape mutations might be more difficult. Additional aspect of interest of PN19 peptide is the potential different target of action compared to that of HR2 peptide. Thus, possible additive inhibitory activity by using such peptides cold be envisioned.

In conclusion, dissecting the IFP region of S2 subunit of SARS-CoV-2 spike glycoprotein, lead us to identify a short amino acid sequence on which inhibitory molecules could be developed. The conservation of several residues in IFP region of S2 spike of SARS-CoV-2 variants and other coronavirus and the role played of its key aromatic residues in viral fusion are relevant. Future investigation will be necessary to develop peptidomimetic compounds mimicking the structure and the side-chain interactions implicated in IFP binding with MPER and membranaceus environment, as potential innovative antiviral treatment to fight SARS-CoV-2 and future coronavirus emergence.

## Authors’ data statement

The authors declare the availability of their data.

## Author contributions

“SG and PR designed the study. MAS, MQ, ADS, LP, FRF and RA performed most of the experiments. MAS, FRF, MQ, SR analyzed the data. MAS, MQ, ADS, RA, SR collected the study data. SG and PR wrote the manuscript. SG, PR, MAS, AMP reviewed the manuscript. SG and PR supervised the study. All authors read and approved the final manuscript.

## Institutional review board statement

Not applicable.

## Informed consent statement

Not applicable.

## Funding

This research was funded by Regione Toscana, Italy, (Bando Ricerca Salute 435, 2018) (to SG).

## Declaration of Competing Interest

The authors declare that they have no known competing financial interests or personal relationships that could have appeared to influence the work reported in this paper.

## Data Availability

Data will be made available on request.

## Appendix Supplementary materials

Image, application 1
