# Supplementary material for: SARS-CoV-2 inhibitory activity of a short peptide derived from internal fusion peptide of S2 subunit of spike glycoprotein
Source: Virus Res. 2023 Jul 15;334:199170. doi: 10.1016/j.virusres.2023.199170 (PMC10384657; doi:10.1016/j.virusres.2023.199170)
Supplement: Supplementary file 1 [file mmc1.docx]

**Supplementary material**

**Analytical characterization of peptides**

Tentagel^®^ S RAM resin was used (loading 0.23 mmol/g). The following Fmoc-amino acid were used: Fmoc-Ala-OH, Fmoc-Cys(Trt)-PH, Fmoc-Asp(tBu)-OH, Fmoc-Glu(tBu)-OH, Fmoc-Phe-OH, Fmoc-Gly-OH, Fmoc-Ile-OH, Fmoc-Lys(Boc)-OH, Fmoc-Leu-OH, Fmoc-Met-OH, Fmoc-Asn(Trt)-OH, Fmoc-Gln(Trt), Fmoc-Arg(Pbf)-OH, Fmoc-Ser(tBu)-OH, Fmoc-Val-OH, Fmoc-Trp(Boc)-OH, Fmoc-Tyr(tBu)-OH. Fmoc deprotections were performed with a solution of 20% piperidine in DMF (2 M). Peptide assembly was performed by repeating the SPPS standard coupling cycle for each amino acid, using Fmoc-protected amino acids (2.5 equiv, 0.4 M in DMF), OxymaPure^®^ (2.5 equiv, 1 M in DMF), and DIC (2.5 equiv, 3 M in DMF). *N*-terminal acetylation was performed using Ac_2_O in DMF for 10 min at room temperature two times. Final cleavage and side-chain deprotections were performed using a mixture of TFA/TIS/H_2_O/EDT (94:3:1.5:1,5, v:v:v:v) at room temperature. After 3 h the resin was filtered off. The peptides were precipitated with cold Et_2_O, centrifuged, and lyophilized. The crude peptides were purified by Reverse-Phase Flash Liquid Chromatography on an Isolera One Flash Chromatography (Biotage, Uppsala, Sweden) using a SNAP Ultra C18 column (12 g) with solvent systems H_2_O (MilliQ) and ACN at 12 mL/min. A second purification step was performed by semipreparative RP-HPLC on a Waters instrument (Separation Module 2695, detector diode array 2996) using a Sepax Bio-C4 column (Sepax Technologies, Newark, USA) (5 μm, 250 × 10 mm) with solvent systems A (0.1% TFA in H_2_O) and B (0.1% TFA in ACN) at 4 mL/min. Analytical characterization of the peptides was performed by HPLC using a Waters ACQUITY HPLC coupled to a single quadrupole ESI-MS (Waters® ZQ Detector, Waters Milford, MA, USA) supplied with a BEH C18 (1.7 μm, 2.1 × 50 mm) column at 35°C, using solvent systems A (0.1% TFA in H_2_O) and B (0.1% TFA in ACN) at 0.6 mL/min.

The crude synthetic peptides were purified by Reverse-Phase Flash Liquid Chromatography (RP-FLC) on an Biotage® Isolera^TM^ One Flash Chromatography (Biotage, Uppsala, Sweden) using a SNAP Ultra C18 column (25 g) at 20 mL/min as solvent systems H_2_O (MilliQ) and ACN (gradient reported in Table 1s). The second step of purification of the peptides was performed by semipreparative RP-HPLC on a Waters instrument (Separation Module 2695, detector diode array 2996) using a Sepax Bio-C18 column (Sepax Technologies, Newark, USA) (5 μm, 250 × 10 mm), at 4 mL/min with solvent systems A (0.1% TFA in H_2_O) and B (0.1% TFA in ACN). Characterization of the peptides was performed by:

1. RP-UHPLC-MS on a Thermo Scientific Ultimate 3000 equipped with a variable wavelength detector and a Thermo Scientific-MSQ PLUS, using a C18 Waters Acquity CSH (130 Å, 1.7 μm, 2.1 × 100 mm) column at 35 °C, at 0.5 mL/min with solvent systems A (0.1% TFA in H2O) and B (0.1% TFA in ACN).
2. HPLC using a Waters ACQUITY HPLC coupled to a single quadrupole ESI-MS (Waters® ZQ Detector, Waters Milford, MA, USA) supplied with a BEH C18 (1.7 μm 2.1× 50 mm) column at 35 °C, at 0.6 mL/min with solvent systems A (0.1% TFA in H2O) and B (0.1% TFA in ACN).

| **Table 1s:** Analytical characterization of the synthetic peptides. | | | |
| --- | --- | --- | --- |
| ***Fragment*** | ***HPLC^a^ gradient (% B)^c^***  ***R_f_ (min)*** | ***HPLC purity (%)*** | ***ESI-MS (m/z) found^e^ (calcd)*** |
| **P23** | 5-95, 4.01 min | 95 | 855.5 (855.3) |
| **PN21** | 20-70, 4.14 min | 95 | 806.9 (807.3) |
| **PN19** | 20-70, 3.90 min | 95 | 1066.8 (1066.8)^f^ |
| **PN17** | 20-70, 4.11 min | >95 | 964.8 (964.7)^f^ |
| **PC21** | 30-90, 3.25 min | 95 | 1130.6 (1130.8)^f^ |
| **PC19** | 30-90, 3.12 min | >95 | 1013.7 (1013.7)^f^ |
| **PC17** | 5-95, 4.31 min | 90 | 883.9 (884.0)^f^ |
| **PNC19** | 30-90, 3.47 min | >95 | 1058.3 (1058.8)^f^ |
| **PNC15** | 20-70, 4,25 min | >95 | 797.8 (798.0)^f^ |
| **PN13** | 30-90^b^, 3.93 min | >95 | 829.6 (829.5)^f^ |
| **PN9** | 30-90^b^, 3.30 min | >95 | 1206.2 (1206.5)^g^ |
| **PN19_F_*_→_*_A888_** | 30-90^b^, 3.27 min | >95 | 1028.9 (1028.7)^f^ |
| **PN19_L_*_→_*_A894_** | 30-90^b^, 3.35 min | >95 | 1045.9 (1045.7)^f^ |
| **PN19_Q_*_→_*_A895_** | 30-90, 3.55 min | >95 | 1037.7 (1038.3)^f^ |
| **PN19_I_*_→_*_A896_** | 30-90^b^, 3.28 min | >95 | 1045.9 (1045.7)^f^ |
| **PN19_P_*_→_*_A897_** | 30-90^d^, 7.38min | 90 | 1053.2 (1053.8)^f^ |
| **PN19_F_*_→_*_A898_** | 30-90^b^, 3.30 min | >95 | 1029.0 (1028.7)^f^ |
| **PN19_M_*_→_*_A900_** | 30-90, 2.93 min | >95 | 1036.3 (1036.7)^f^ |
| **PN19_Q_*_→_*_A901_** | 30-90, 3.38 min | 95 | 1038.2 (1038.3)^f^ |
| **PN19_M_*_→_*_A902_** | 30-90, 2.92 min | 93 | 1036.7 (1036.7)^f^ |
| **PN19_Y_*_→_*_A904_** | 30-90, 3.16 min | 95 | 1020.3 (1020.7)^f^ |
| **PN19_R_*_→_*_A905_** | 30-90^b^, 4.27 min | 93 | 1024.2 (1024.2)^f^ |
| **PN19_F_*_→_*_A906_** | 30-90^b^, 3.62 min | >95 | 1028.9 (1028.7)^f^ |
| **MPER25** | 40-90^b^, 4.08 min | 93 | 1078.3 (1077.9) |
| **MPER25c** | 40-90^b^, 4.87 min | >95 | 1083.5 (1083.3) |
| The peptides were characterized by ^a^RP-UHPLC-MS on a Thermo Scientific Ultimate 3000 equipped with a variable wavelength detector and a Thermo Scientific-MSQ PLUS, using a C18 Waters Acquity CSH (130 Å, 1.7 μm, 2.1 × 100 mm) column at 35 °C, at 0.5 mL/min. ^b^RP-HPLC Alliance Chromatography system (Waters, Milford Massachusetts, USA) with a BEH C18 (1.7 μm 2.1× 50 mm) column at 35°C, 0.6 mL/min, coupled to a single quadrupole ESI-MS Micromass ZQ (Waters, Milford Massachusetts, USA). Eluents: 0.1% (v/v) TFA in H_2_O (A) and 0.1% (v/v) TFA in ACN (B), λ 215 nm. Gradient times: ^c^5min; ^d^10min. ESI-MS: detected as ^e^[M+3H]^3+^, ^f^[M+2H]^2+^, ^g^[M+H]^+^. | | | |

***P23***

***
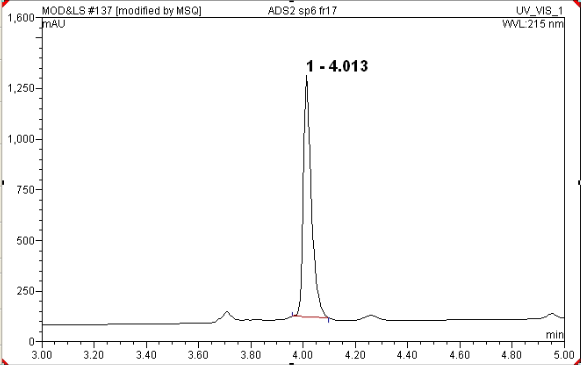
***


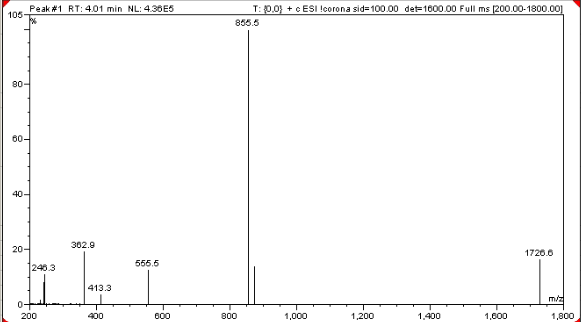


**Figure 1s.** UHPLC chromatogram (upper panel) and MS spectrum (lower panel) of **P23**

***PN21***

***
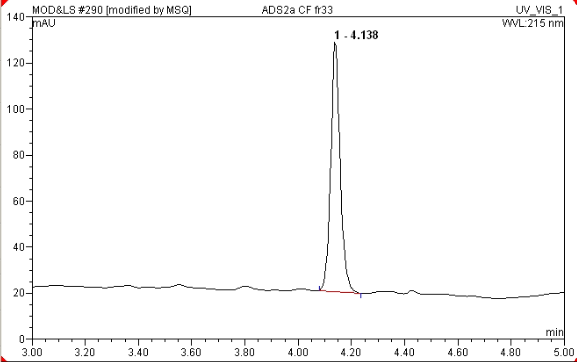
***


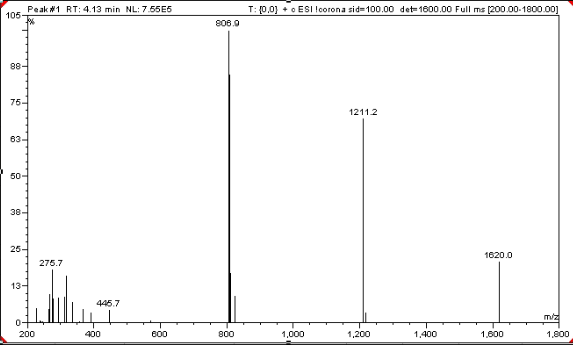


**Figure 2s.** UHPLC chromatogram (upper panel) and MS spectrum (lower panel) of **PN21**

***PN19***

***
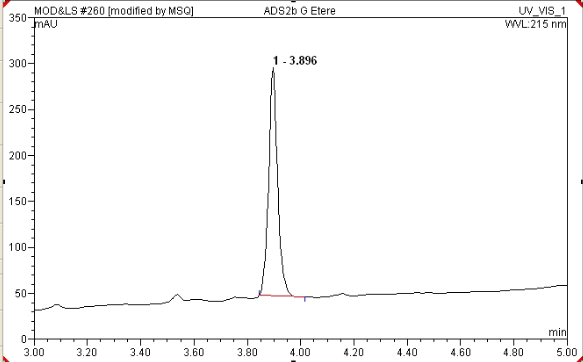
***


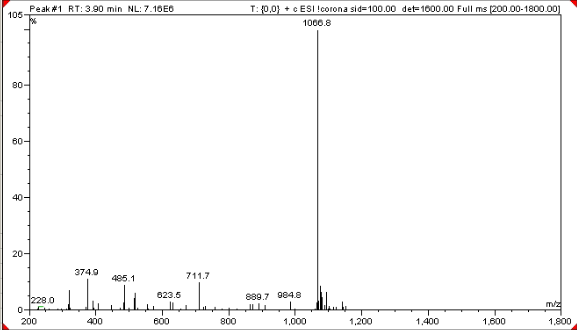


**Figure 3s.** UHPLC chromatogram (upper panel) and MS spectrum (lower panel) of **PN19**

***PN17***

***
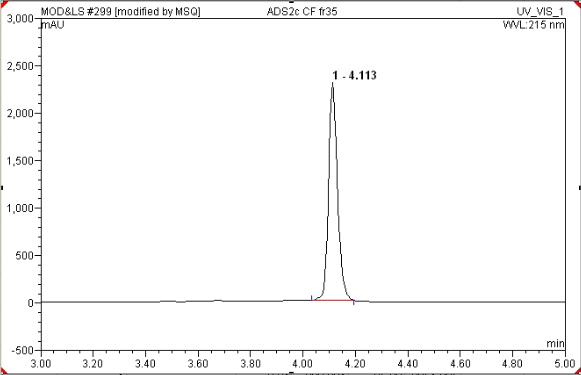
***


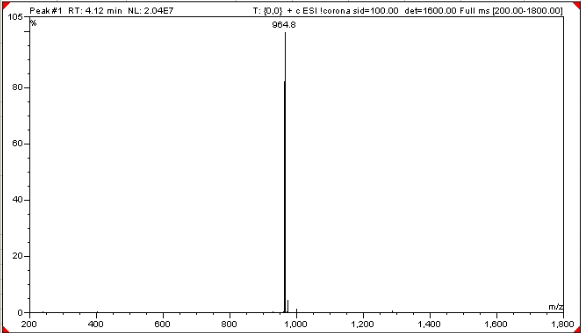


**Figure 4s.** UHPLC chromatogram (upper panel) and MS spectrum (lower panel) of **PN17**

***PC21***

***
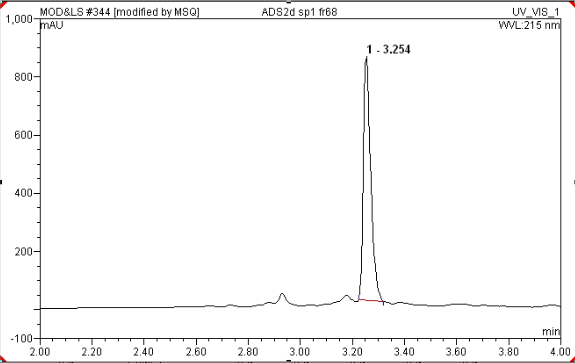
***


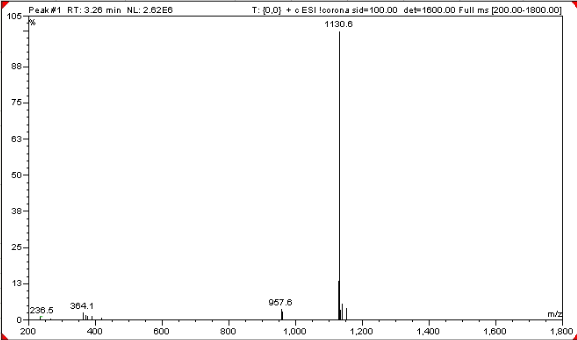


**Figure 5s.** UHPLC chromatogram (upper panel) and MS spectrum (lower panel) of **PC21**

***PC19***

***
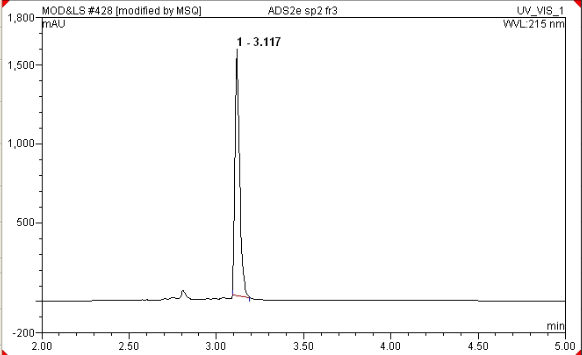
***


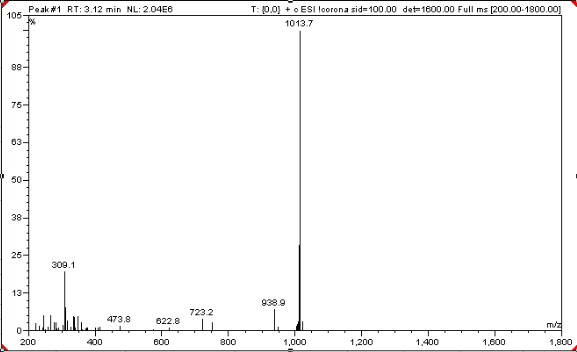


**Figure 6s.** UHPLC chromatogram (upper panel) and MS spectrum (lower panel) of **PC19**

***PC17***

***
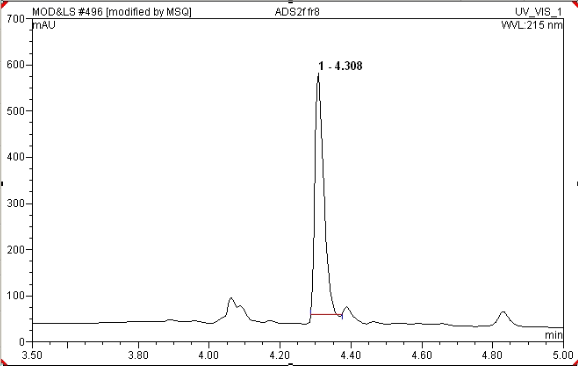
***


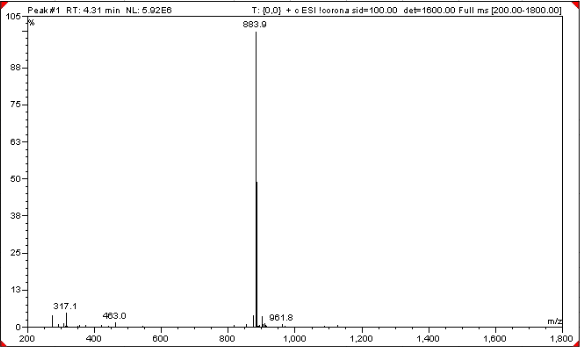


**Figure 7s.** UHPLC chromatogram (upper panel) and MS spectrum (lower panel) of **PC17**

***PNC19***

***
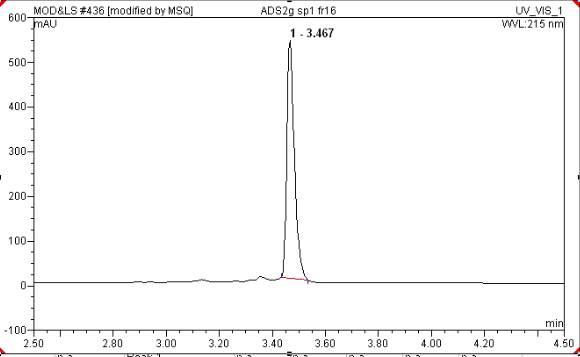
***


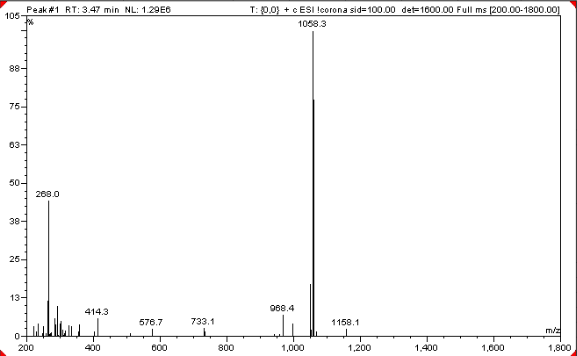


**Figure 8s.** UHPLC chromatogram (upper panel) and MS spectrum (lower panel) of **PNC19**

***PNC15***

***
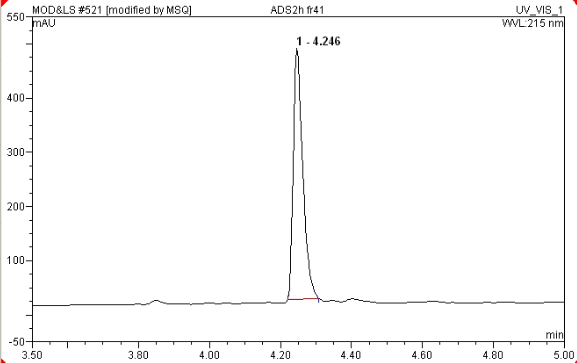
***


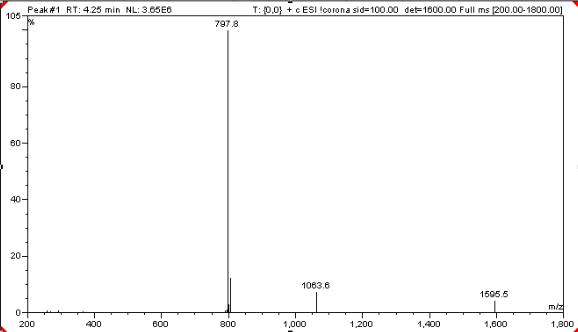


**Figure 9s.** UHPLC chromatogram (upper panel) and MS spectrum (lower panel) of **PNC15**

***PN13***

***
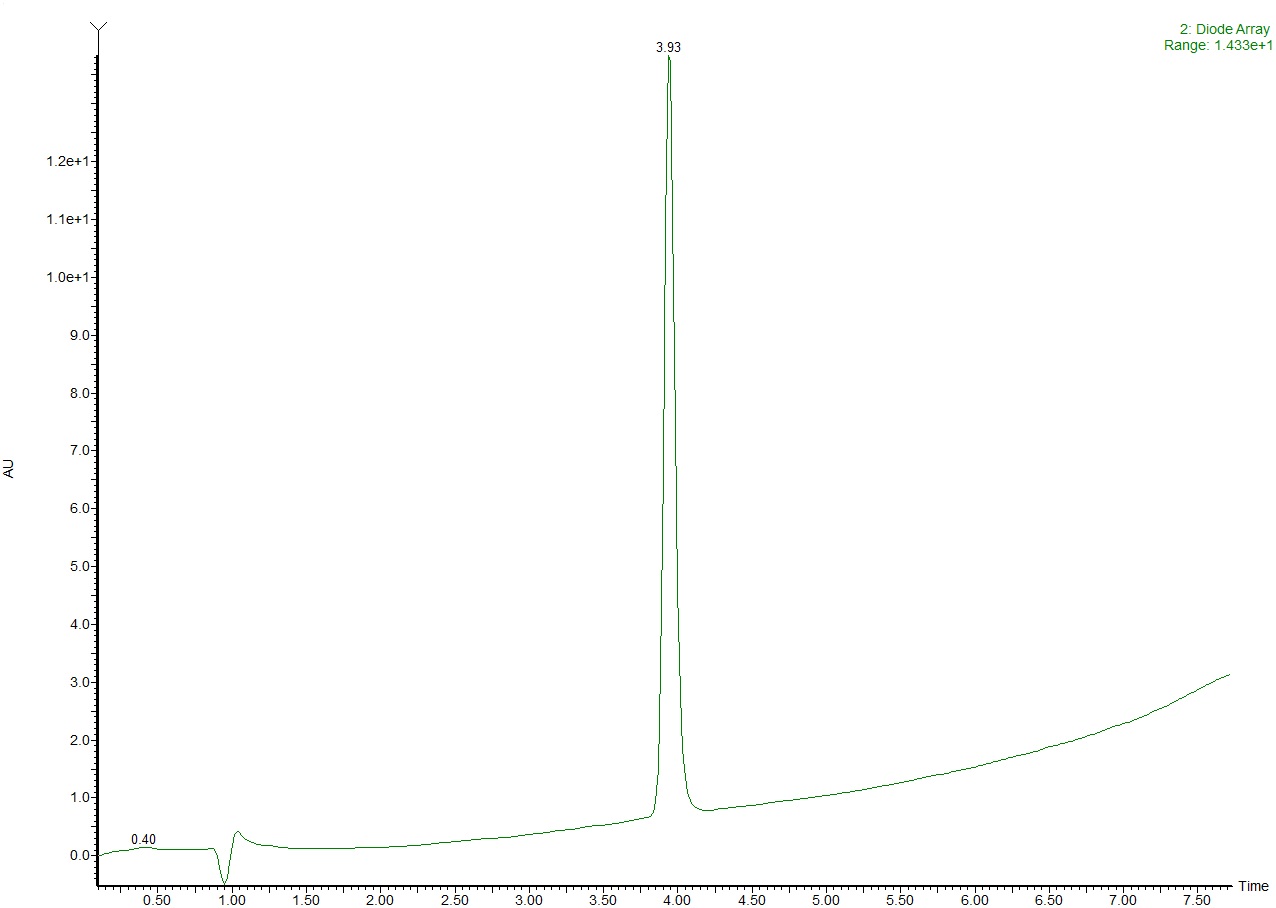
***

***
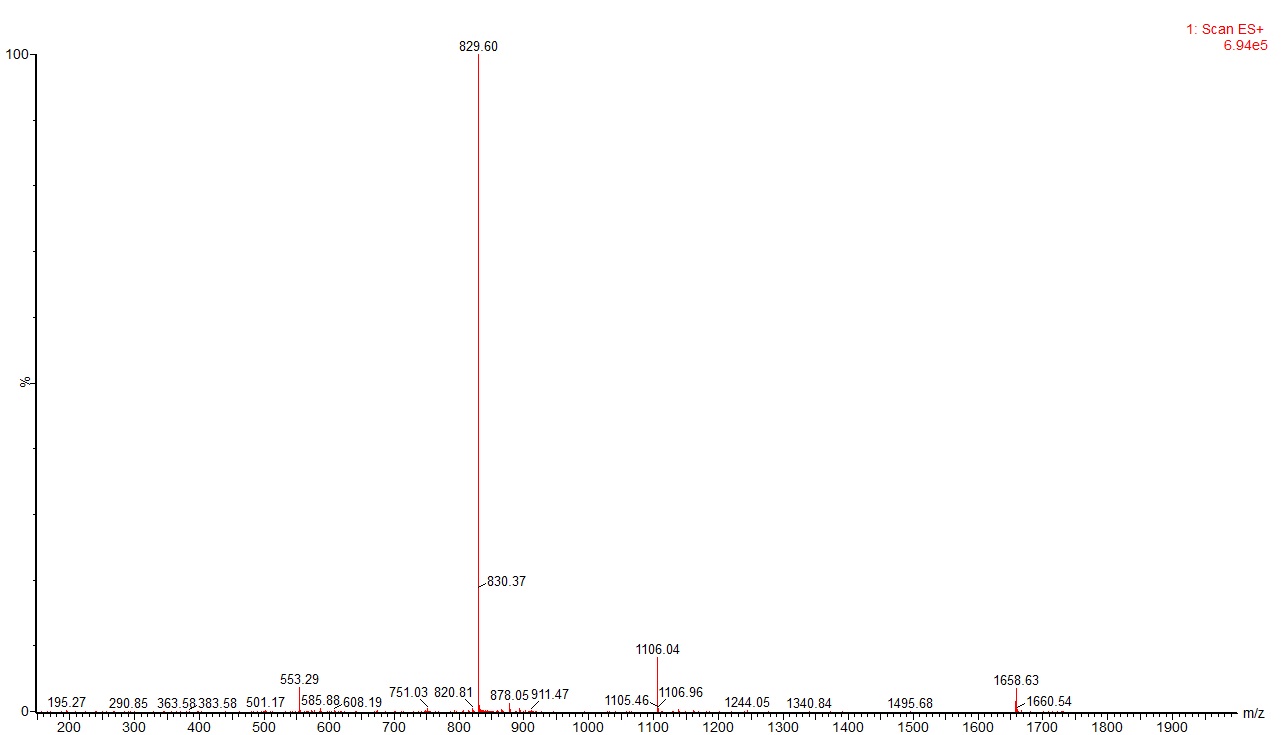
***

**Figure 10s.** HPLC chromatogram (upper panel) and MS spectrum (lower panel) of **PN13**

***PN9***

***
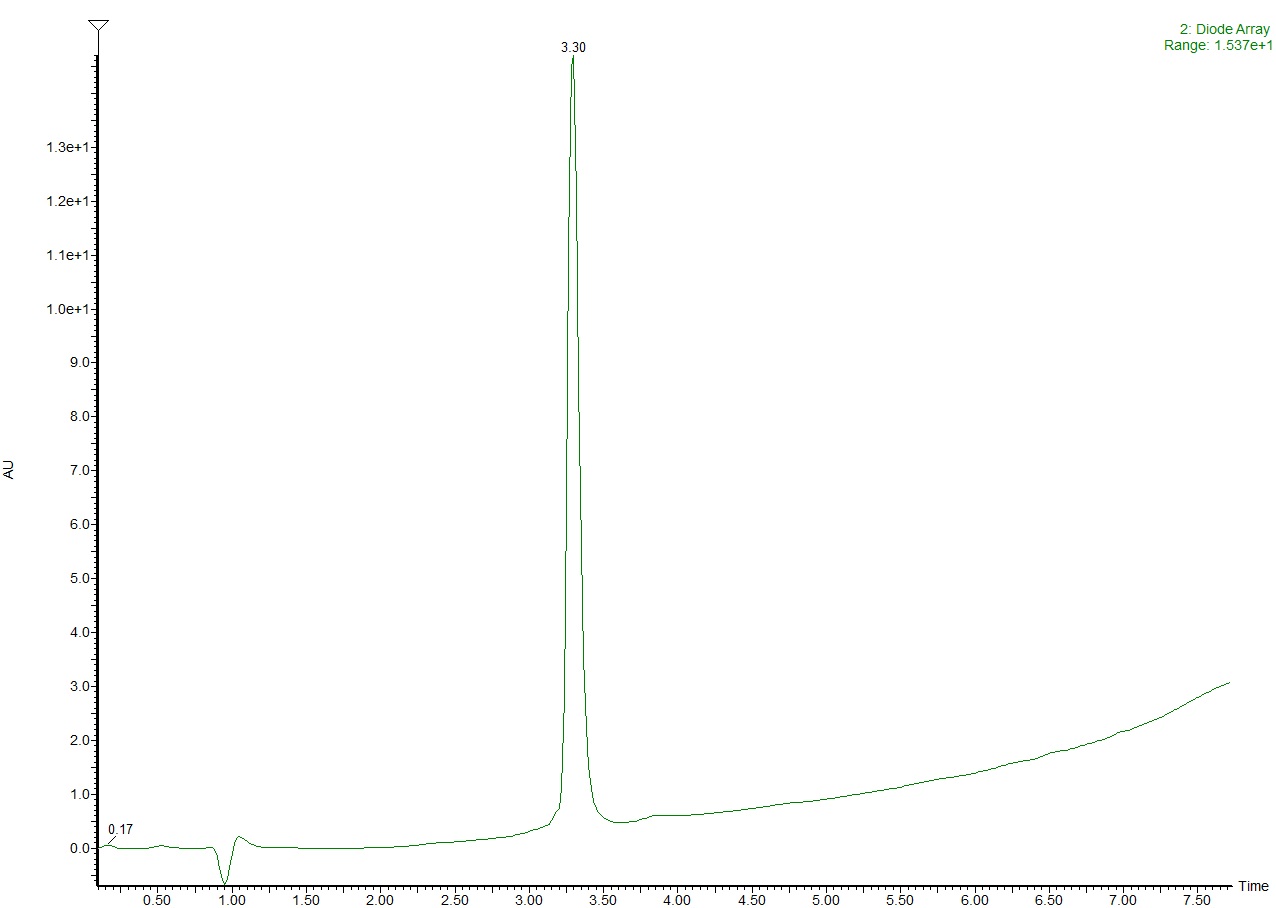
***

***
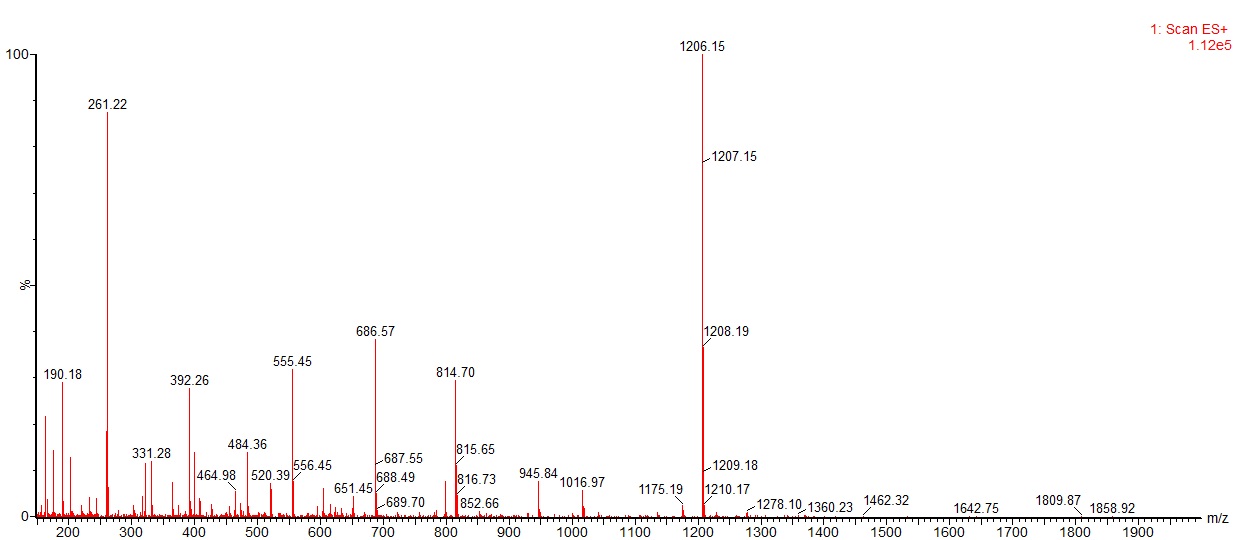
***

**Figure 11s.** HPLC chromatogram (upper panel) and MS spectrum (lower panel) of **PN9**

***PN19_F→A888_***

***
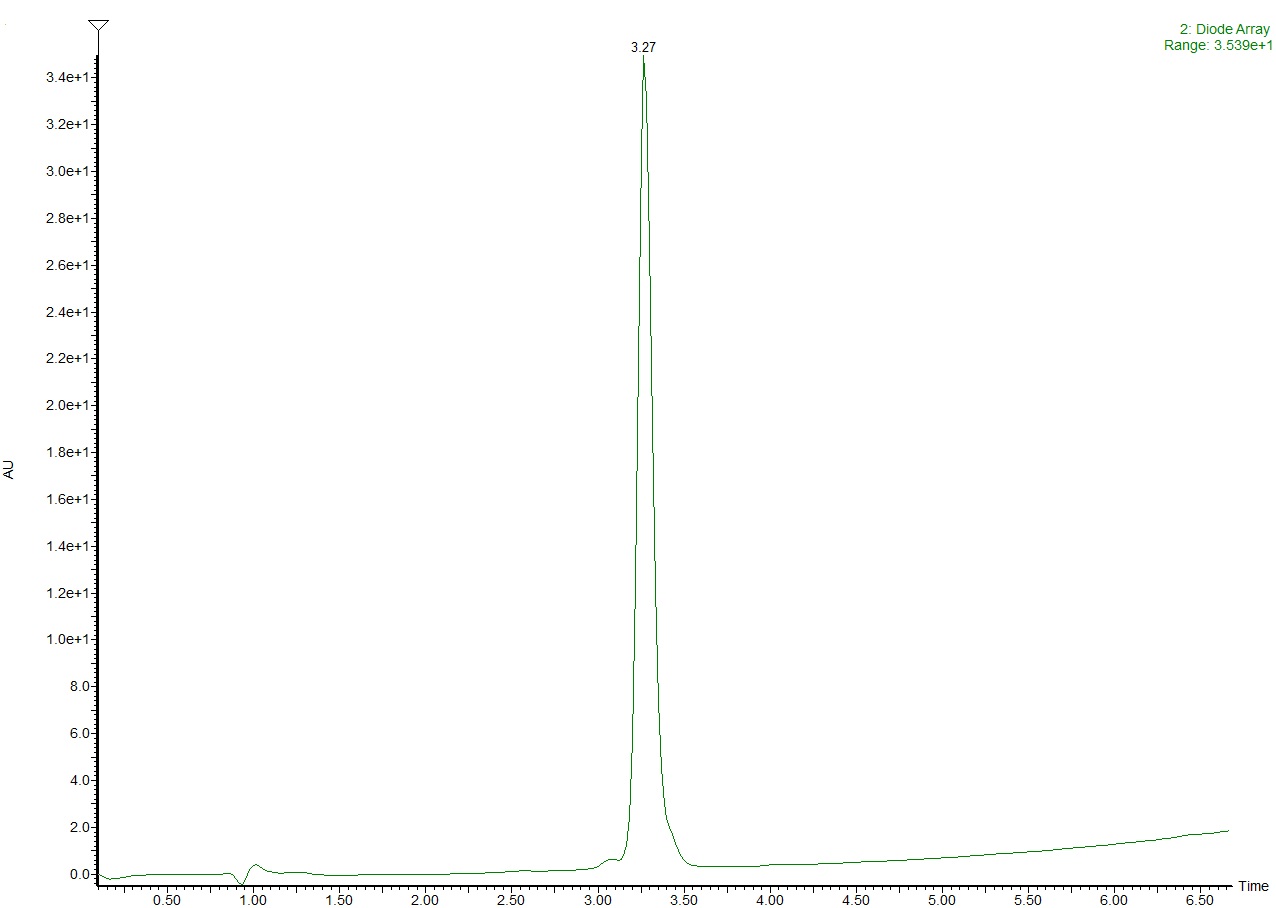
***

***
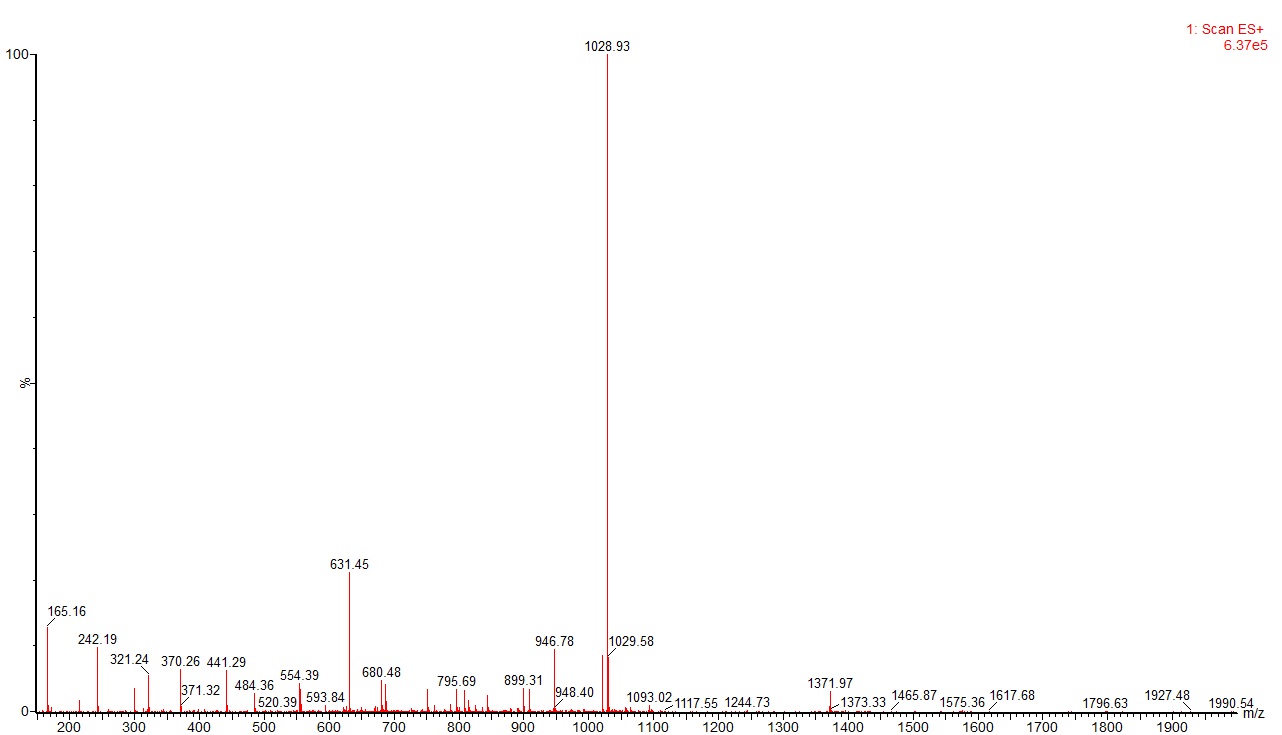
***

**Figure 12s.** HPLC chromatogram (upper panel) and MS spectrum (lower panel) of **PN19_F→A888_**

***PN19_L→A894_***

***
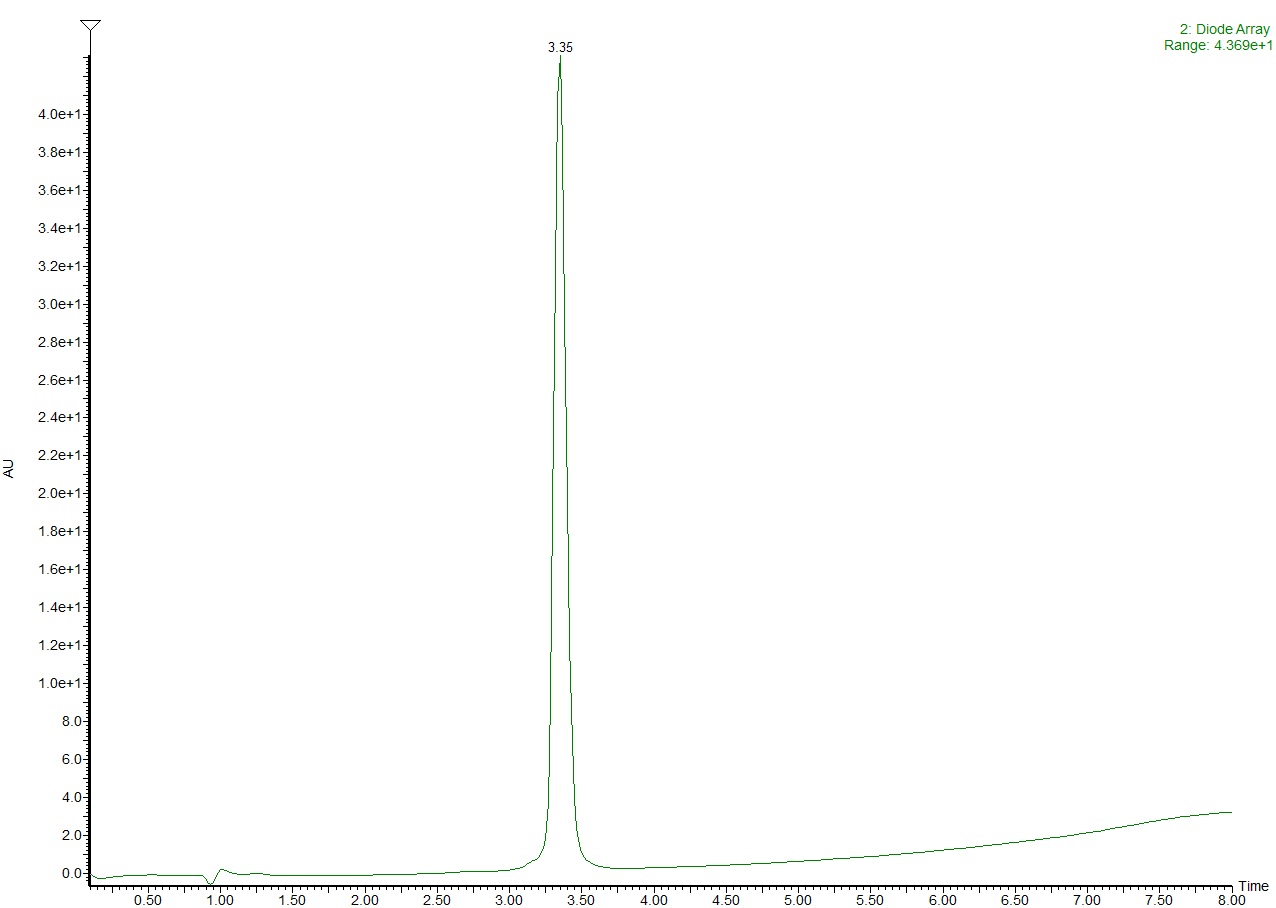
***

***
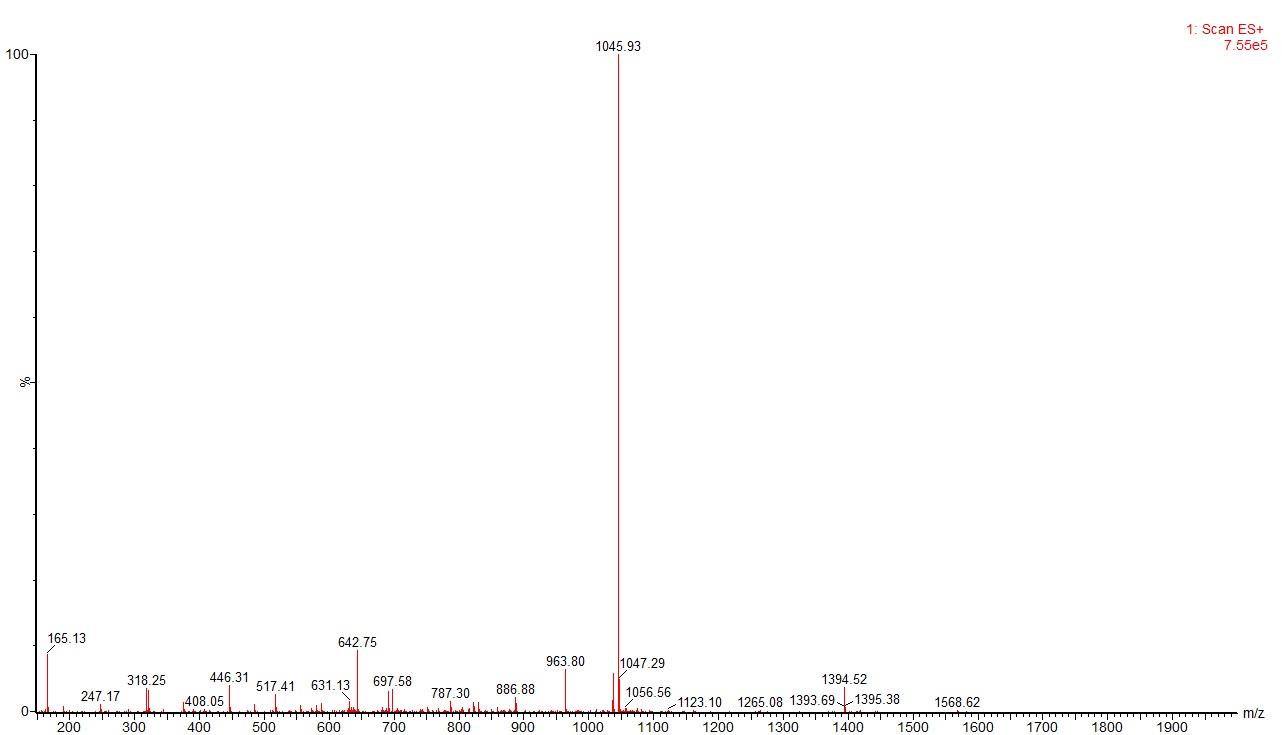
***

**Figure 13s.** HPLC chromatogram (upper panel) and MS spectrum (lower panel) of **PN19_L→A894_**

***PN19_Q→A895_***

***
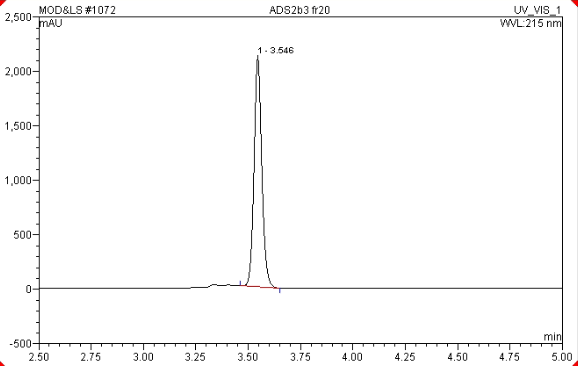
***


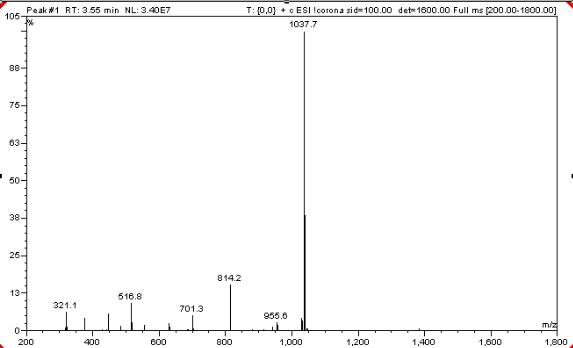


**Figure 14s.** UHPLC chromatogram (upper panel) and MS spectrum (lower panel) of **PN19_Q→A895_**

***PN19_I→A896_***

***
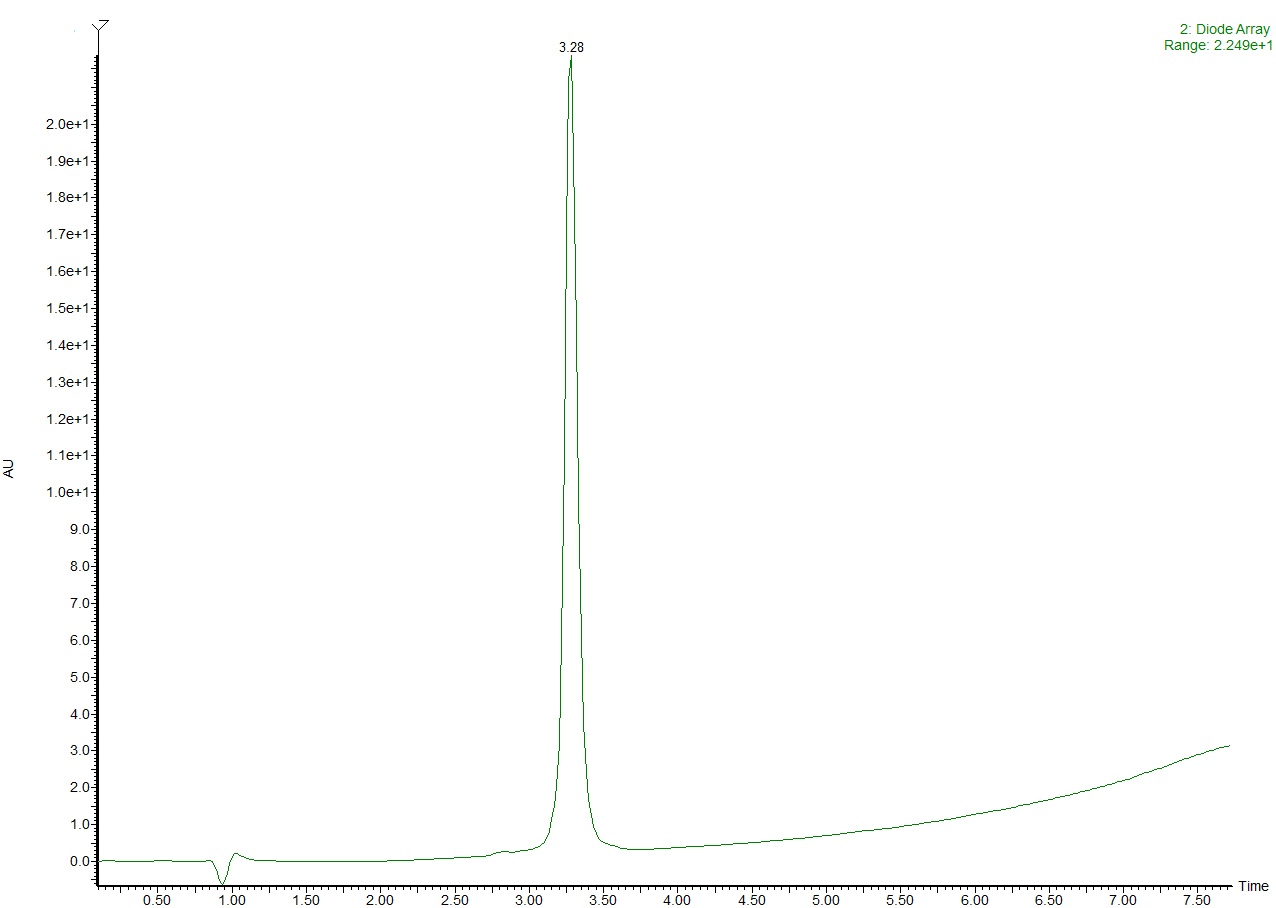
***

***
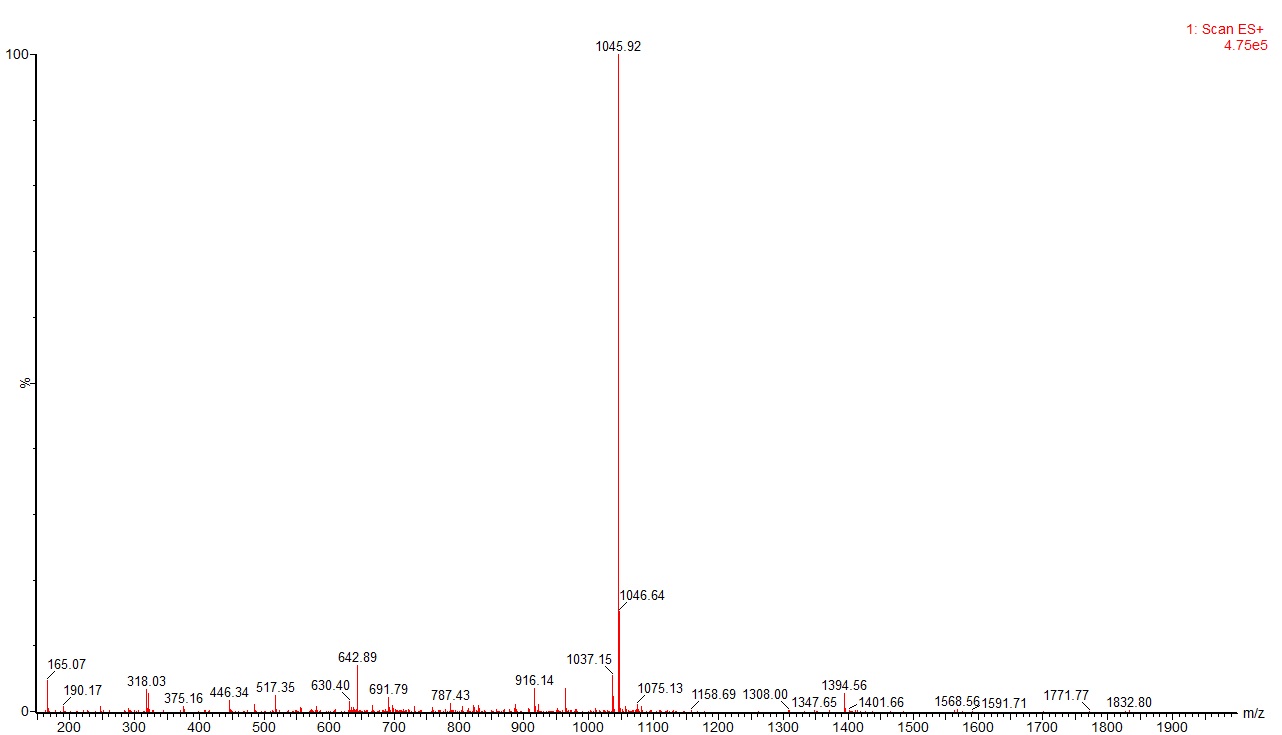
***

**Figure 15s.** HPLC chromatogram (upper panel) and MS spectrum (lower panel) of **PN19_I→A896_**

***PN19_P→A897_***

***
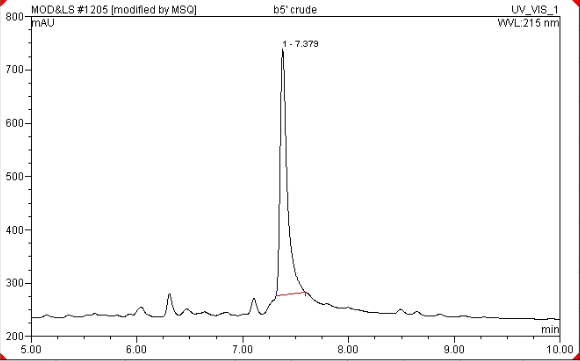
***

***
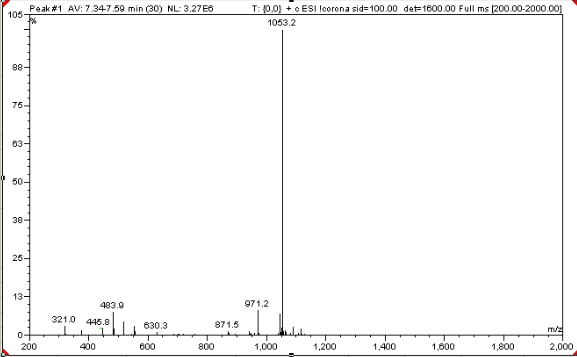
***

**Figure 16s.** UHPLC chromatogram (upper panel) and MS spectrum (lower panel) of **PN19_P→A897_**

***PN19_F→A898_***

***
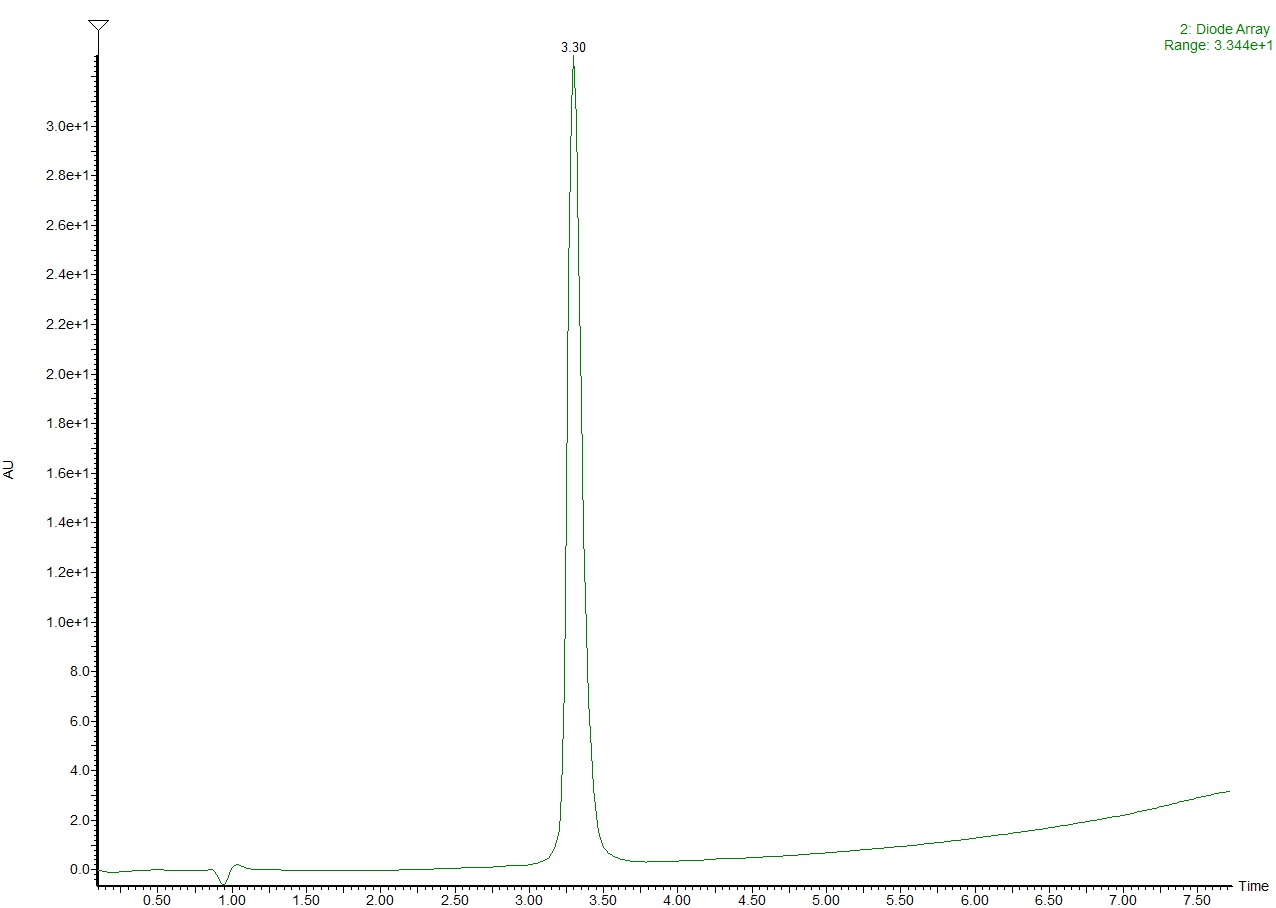
***

***
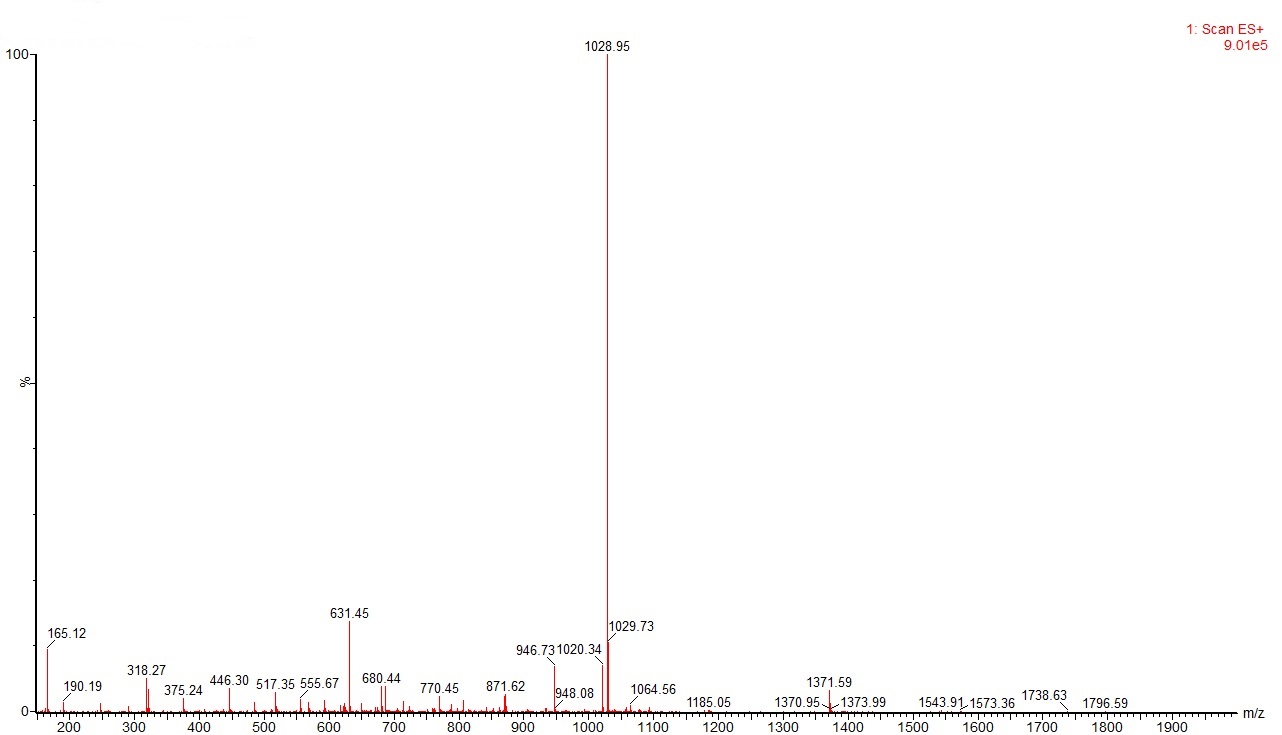
***

**Figure 17s.** HPLC chromatogram (upper panel) and MS spectrum (lower panel) of **PN19_F→A898_**

***PN19_M→A900_***

***
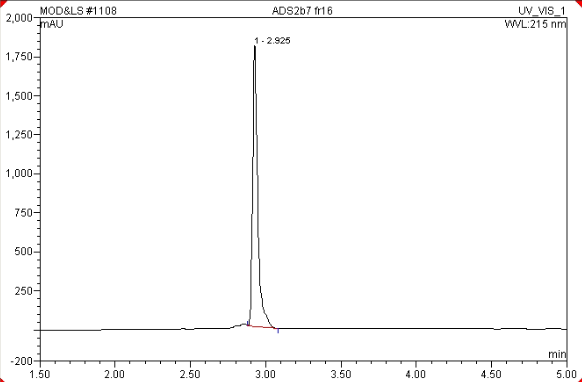
***


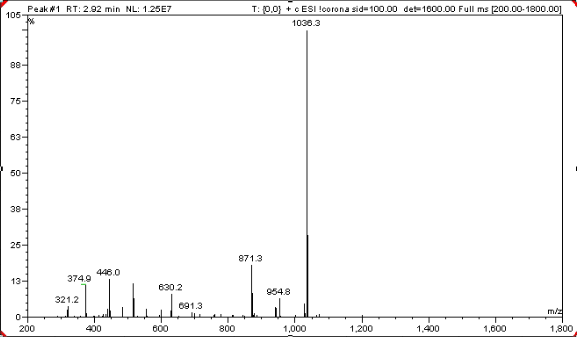


**Figure 18s.** UHPLC chromatogram (upper panel) and MS spectrum (lower panel) of **PN19_M→A900_**

***PN19_Q→A901_***

***
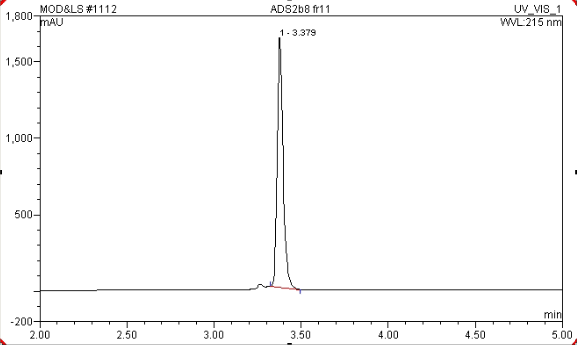
***


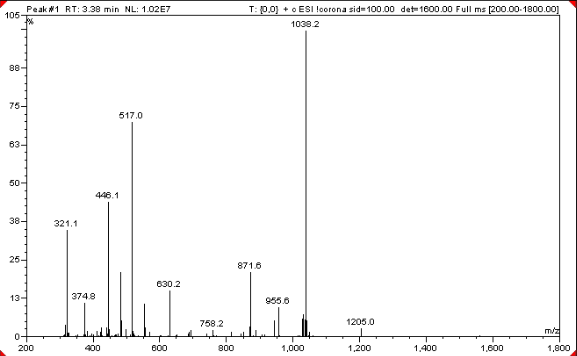


**Figure 19s.** UHPLC chromatogram (upper panel) and MS spectrum (lower panel) of **PN19_Q→A901_**

***PN19_M→A902_***

***
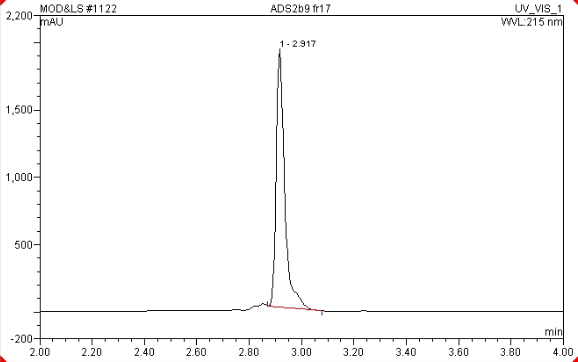
***


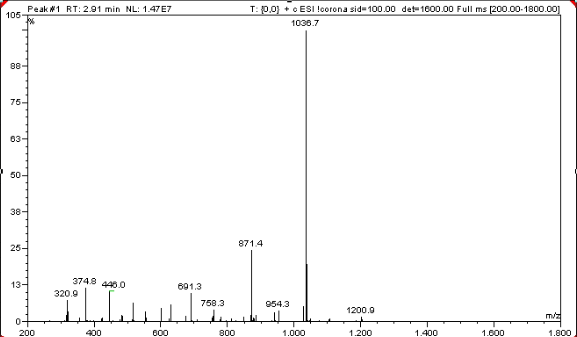


**Figure 20s.** UHPLC chromatogram (upper panel) and MS spectrum (lower panel) of **PN19_M→A902_**

***PN19_Y_******_→A904_***

***
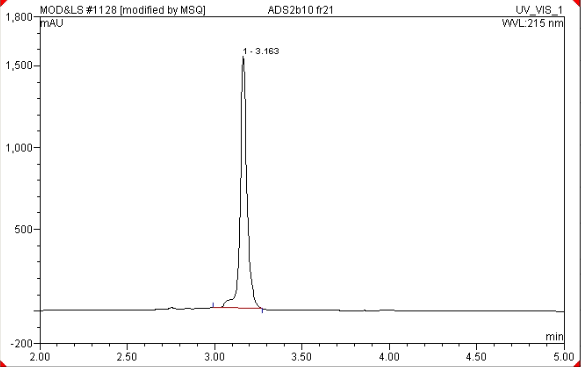
***


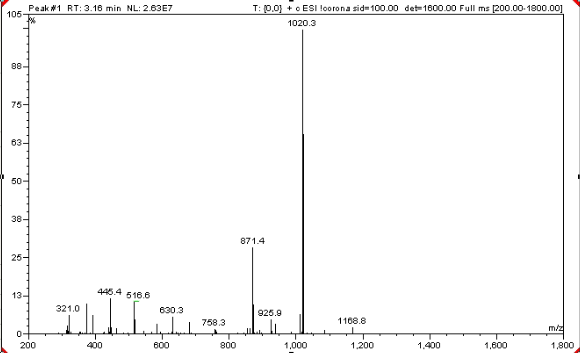


**Figure 21s.** UHPLC chromatogram (upper panel) and MS spectrum (lower panel) of **PN19_Y→A904_**

***PN19_R→A905_***

***
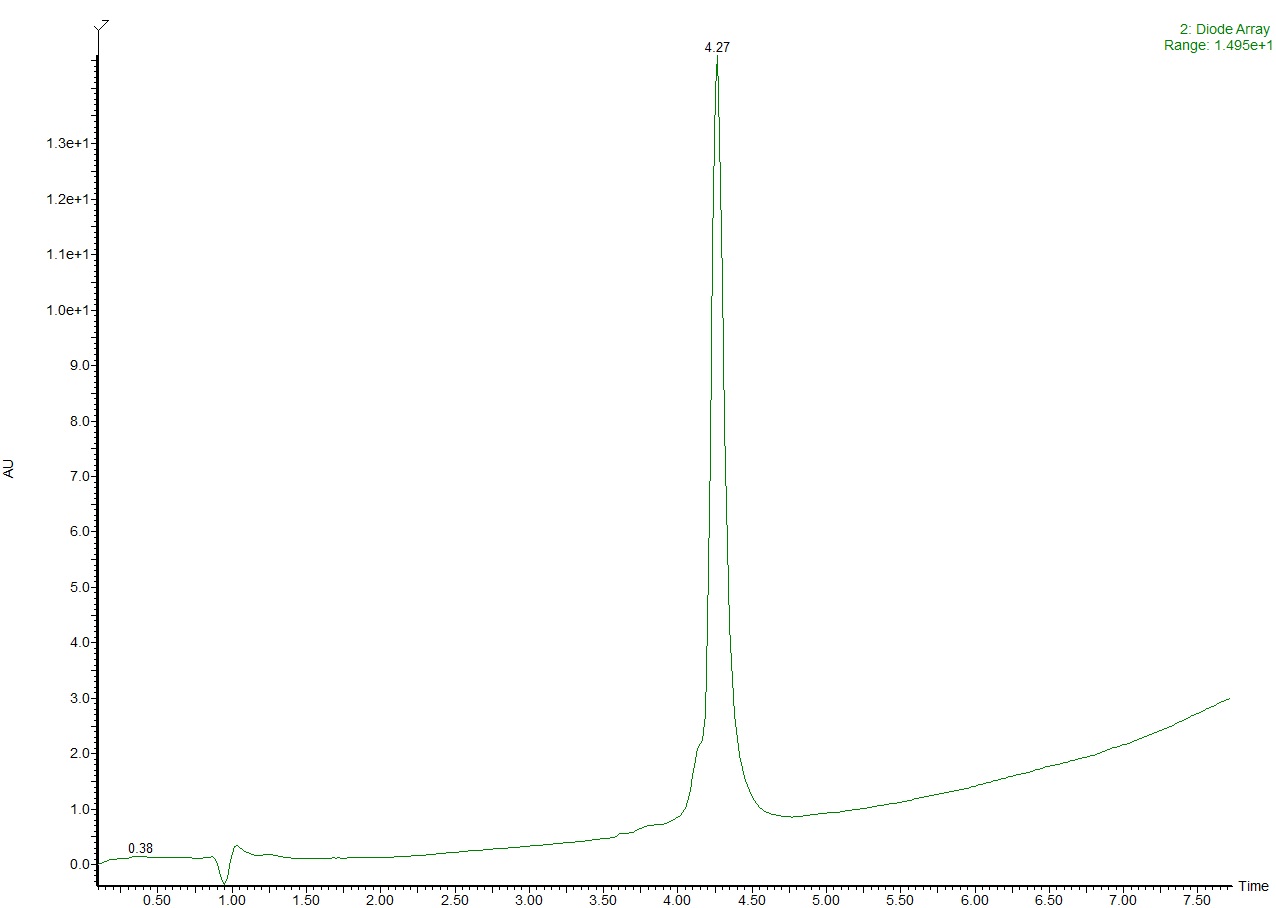
***


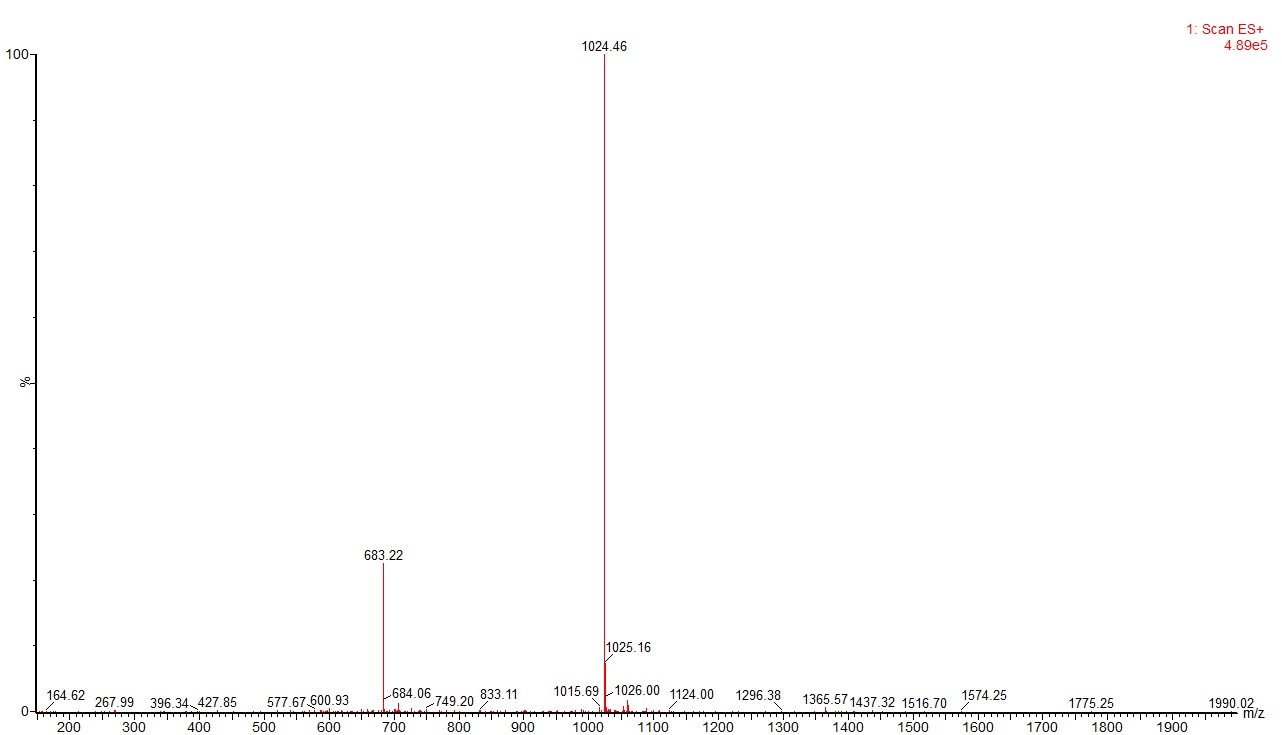


**Figure 22s.** HPLC chromatogram (upper panel) and MS spectrum (lower panel) of **PN19_R→A905_**

***PN19_F→A906_***

***
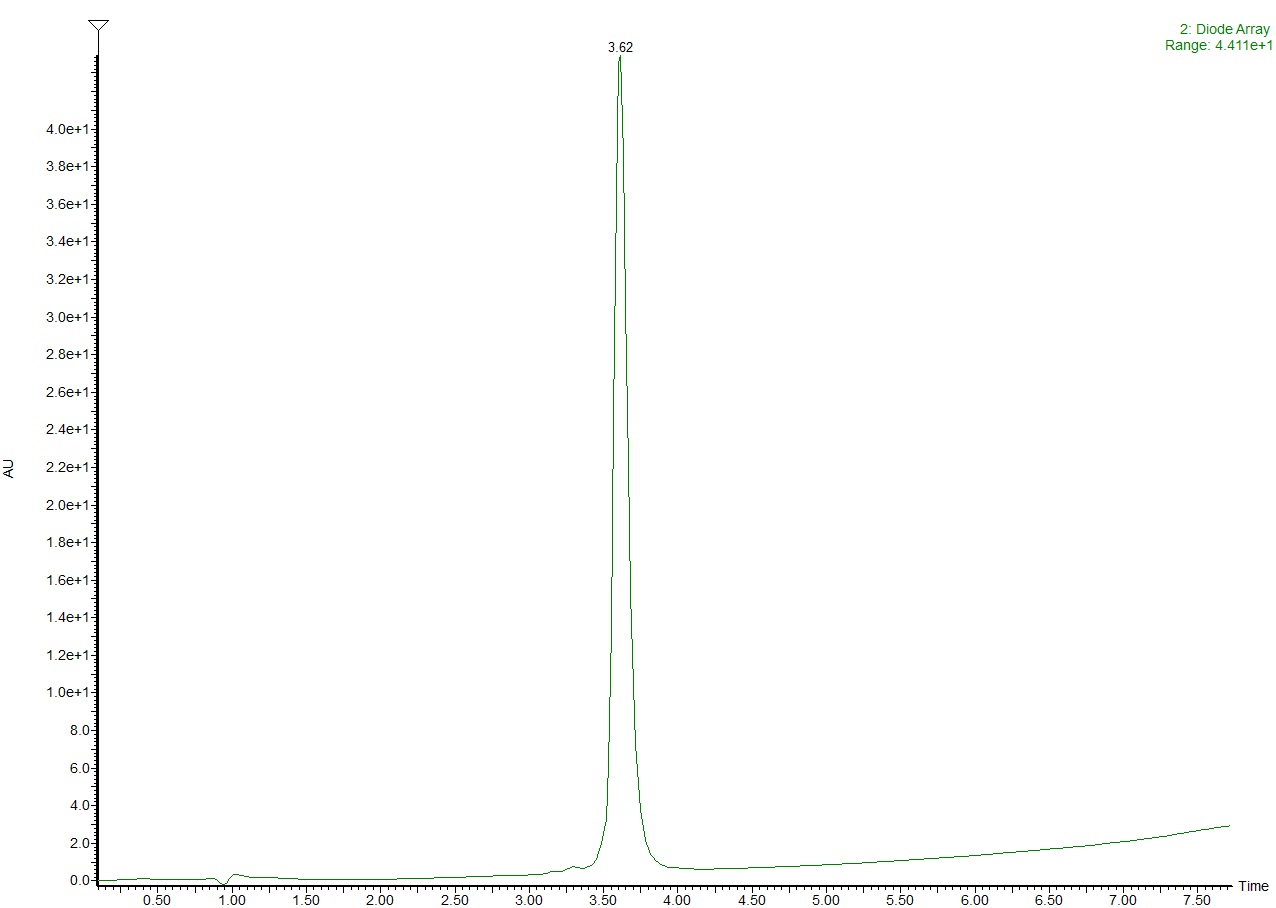
***


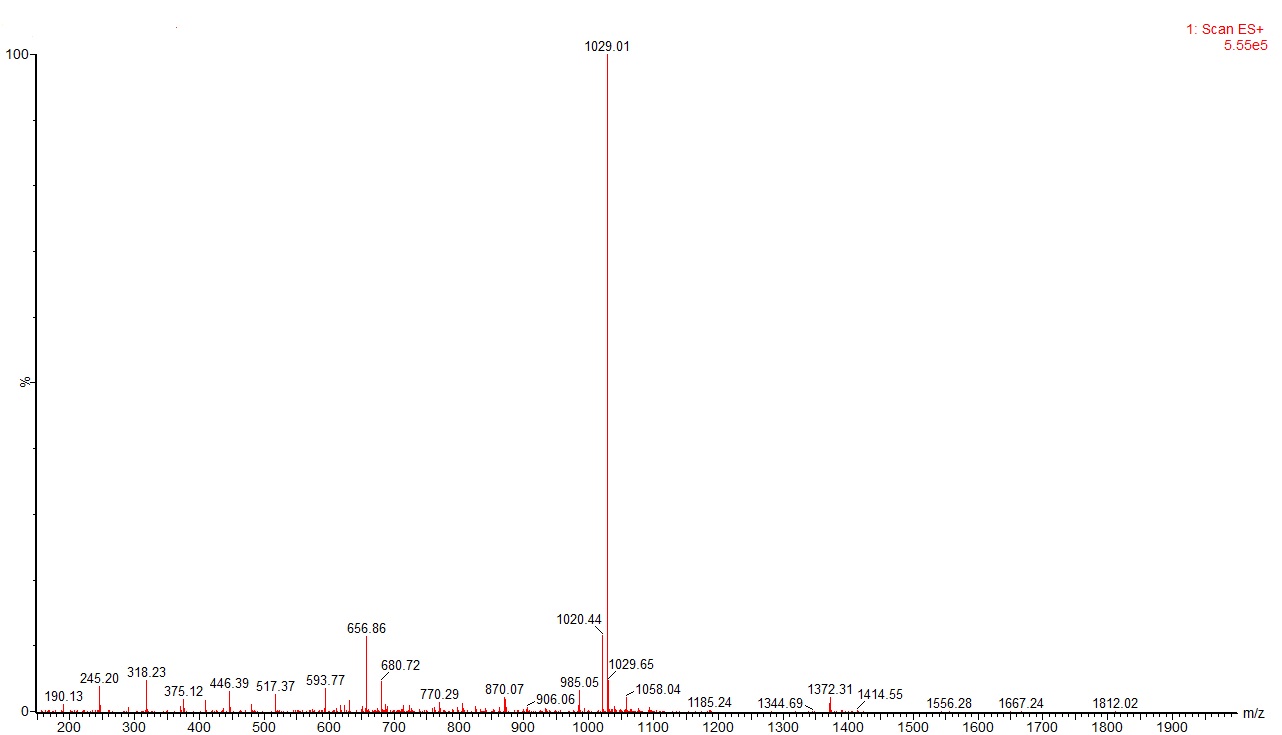


**Figure 23s.** HPLC chromatogram (upper panel) and MS spectrum (lower panel) of **PN19_F→A906_**

***MPER25***

***
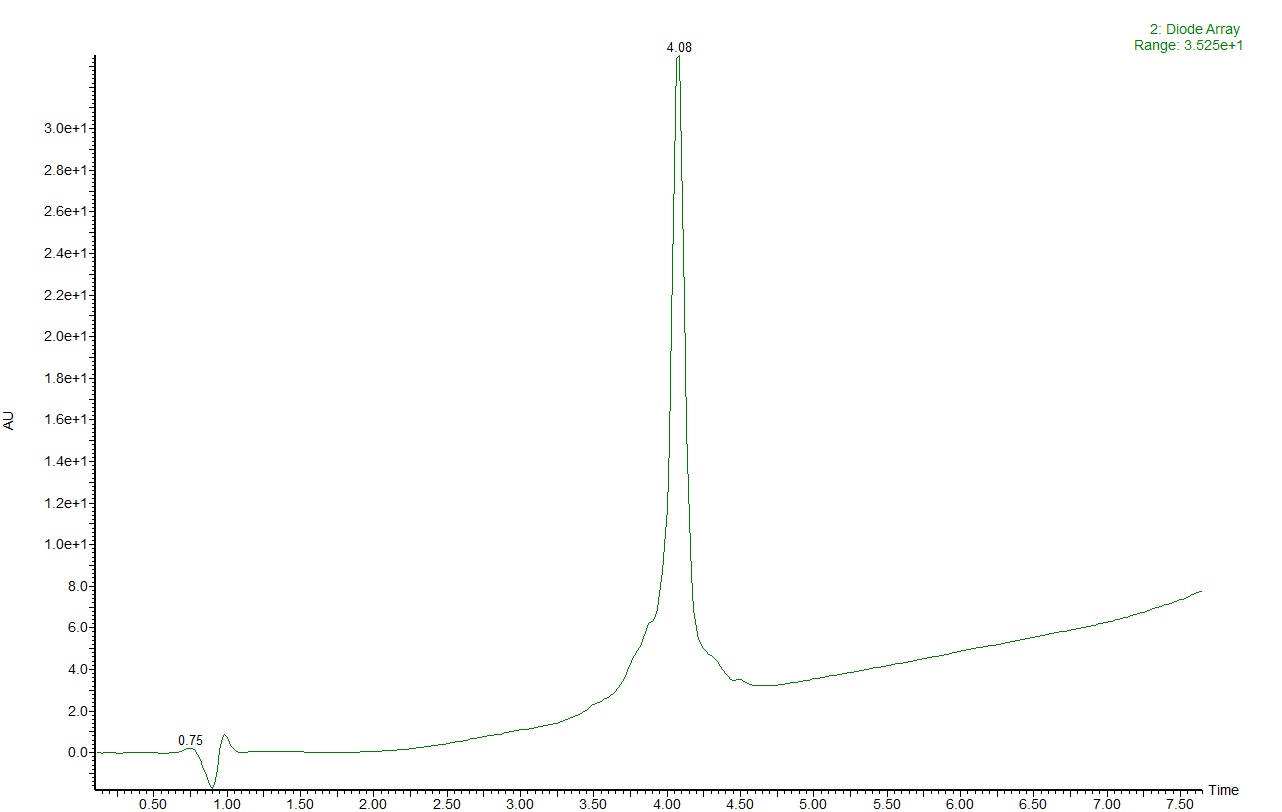
***


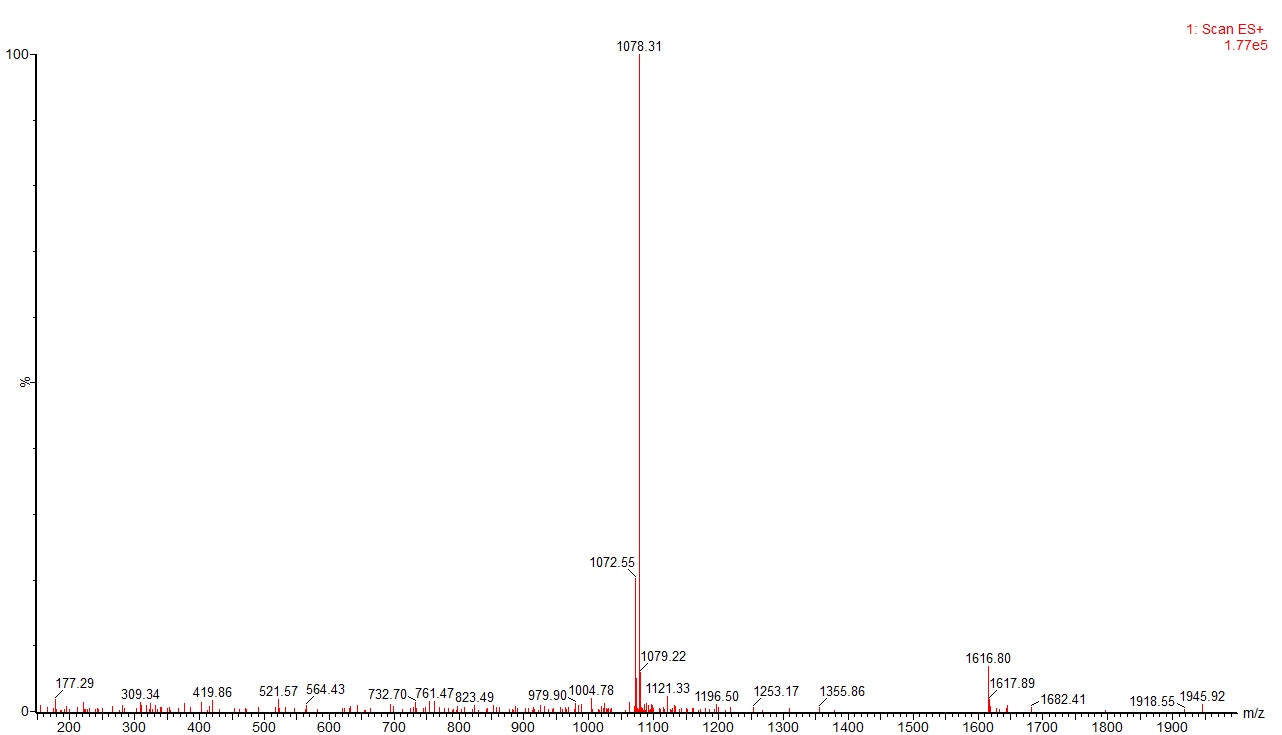


**Figure 24s.** HPLC chromatogram (upper panel) and MS spectrum (lower panel) of **MPER25**

***MPER25c***

***
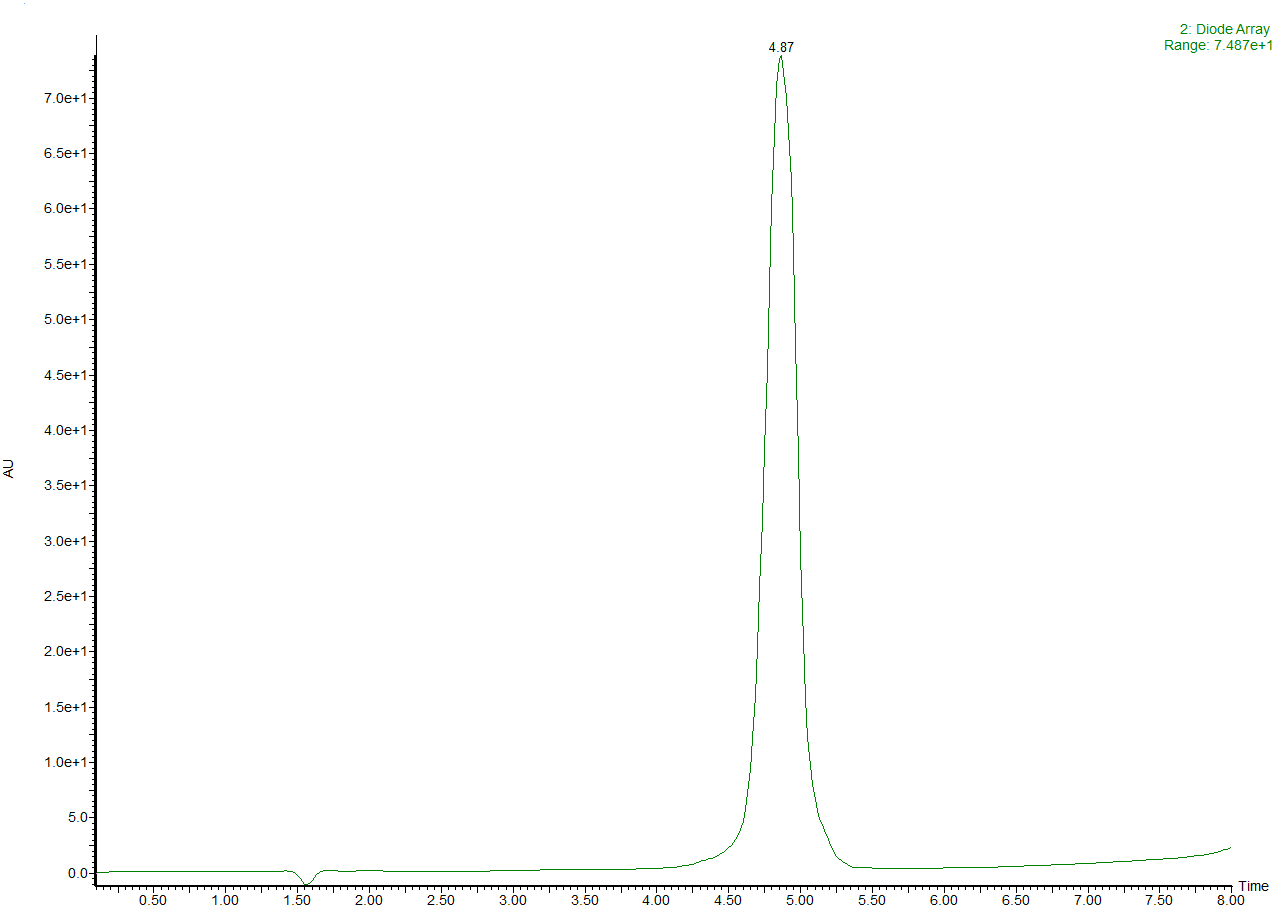
***


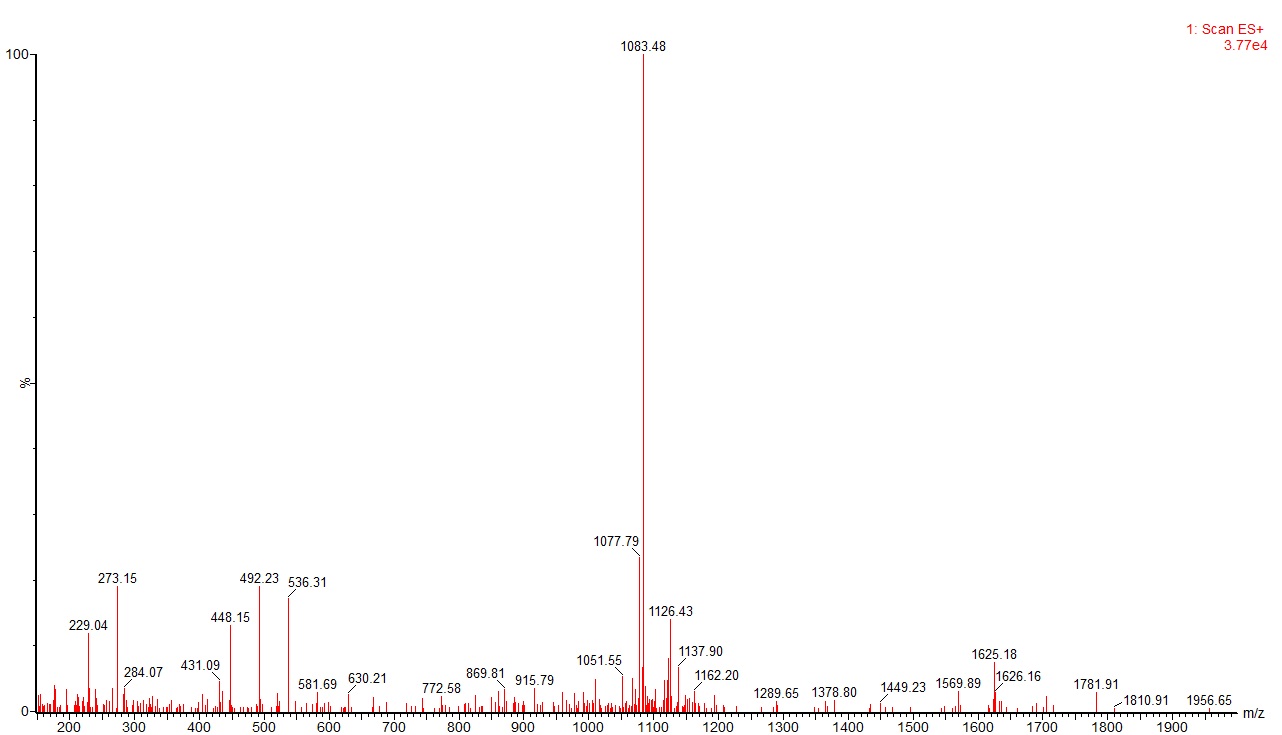


**Figure 25s.** HPLC chromatogram (upper panel) and MS spectrum (lower panel) of **MPER25c**

*Surface plasmon resonance (SPR) experiments.*

Experiments were performed using the Biacore® X100 instrument from Cytiva (Milano, Italy). All binding analyses were made at 25ºC using HBS-EP+ as running buffer. *Peptide Immobilization.* Peptide MPER25c was covalently immobilized on the sensor chip CM5-type surface according to the general thiol coupling strategy. The immobilized MPER25c peptide raised a final immobilization level of 2800 ± 200 Resonance Units (RU). Reference channel 1 was activated and then was directly blocked with cysteine solution and used as blank to remove the non-specific signal depending on interactions between molecules present in the samples and gold on sensor chip surface. *SPR Binding Experiments.* Peptides PN19, PN19_F🡒A898_, PN19_Y🡒A904_, PN19_F🡒A906_ (1 mg/mL stock solutions) were prepared in water containing 30% acetonitrile. Peptides were then independently flowed at 100 µM over the immobilized MPER25c peptide during 120 sec. Interactions were recorded as separate sensorgrams. Responses were measured in Resonance Units (RU) as the difference between active and reference channel. Surface was regenerated with a 20 sec pulse of 50 mM Glycine-HCl pH 2.5 followed by two injections of a 15 sec pulse of a solution 10 mM NaOH/1M NaCl allowing the complete removal of specifically and non-specifically bounded biological material from the surface. *Kinetic Experiments.* Single cycle kinetic experiments were conducted following the previously described SPR protocol (Real Fernandez et al, 2015; Cimitan et al, 2005). Peptide PN19 was tested at different initial calculated concentrations 200, 100, 50, 25, and 12.5 μM and solutions were injected in duplicate over the immobilized MPER25c peptide for 120 seconds. Running buffer was then flushed for 600 seconds during the dissociation phase and finally the chip surface was regenerated by injecting a glycine solution 50 mM pH 2.5 for 20 seconds and a two injections of sodium hydroxide solution 10 mM containing 1M NaCl for 15 seconds both at a flow rate of 10 μL/min. Kinetic experiments were elaborated with Biacore Evaluation Software 2.0 using channel 2 signals.

**Table 2s.** Spike protein mutation in SARS-CoV-2 isolate variants used in the study

| Isolate variant | WHO label | Pango lineage | Spike protein mutations | | | | | |
| --- | --- | --- | --- | --- | --- | --- | --- | --- |
|  |  |  | S1 | | | |  | S2 |
|  |  |  | NTD | RBD | Furin site | other |  |  |
| SCV2/Fi/3/22 |  | B.1 | none | none | none | D614G |  |  |
| SCV2/Fi/1/21 | Alpha | B.1.1.7 | Δ69-70  Δ144 | N501Y | P681H | A570D  D614G |  | T716I  S982A  D1118H  K1191N |
| SCV2/Fi/2/21 | Delta | B1.617.2 | T19R  G141D  Δ156-157  R158G | L452R  T478K | P681R | D614G |  | D950N |
| SCV2/Fi/1/22 | Omicron | BA.1 |  |  |  |  |  |  |


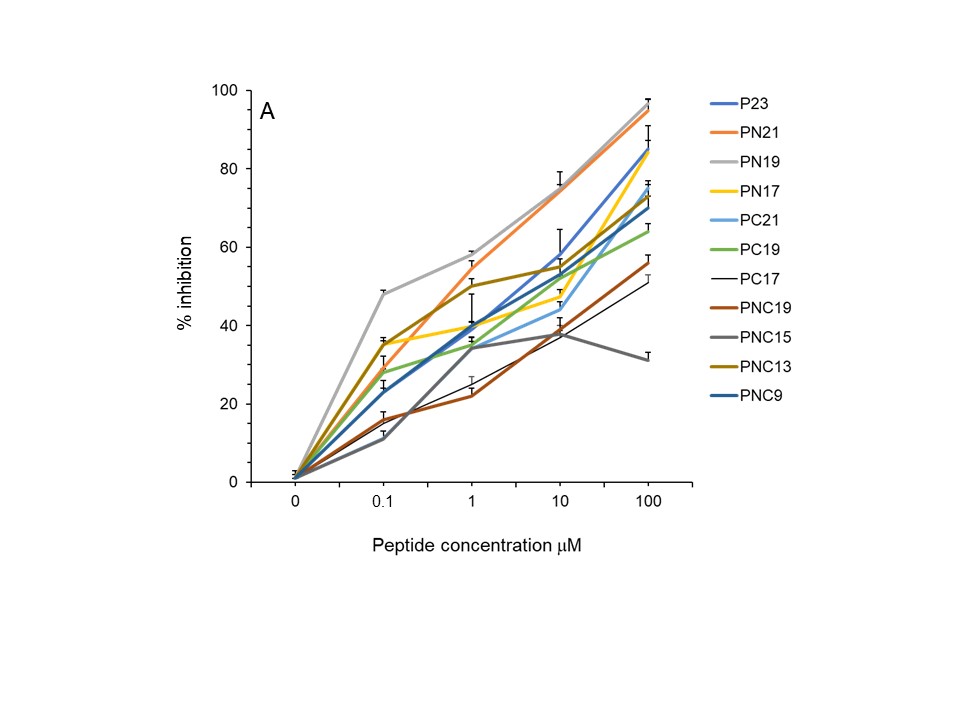

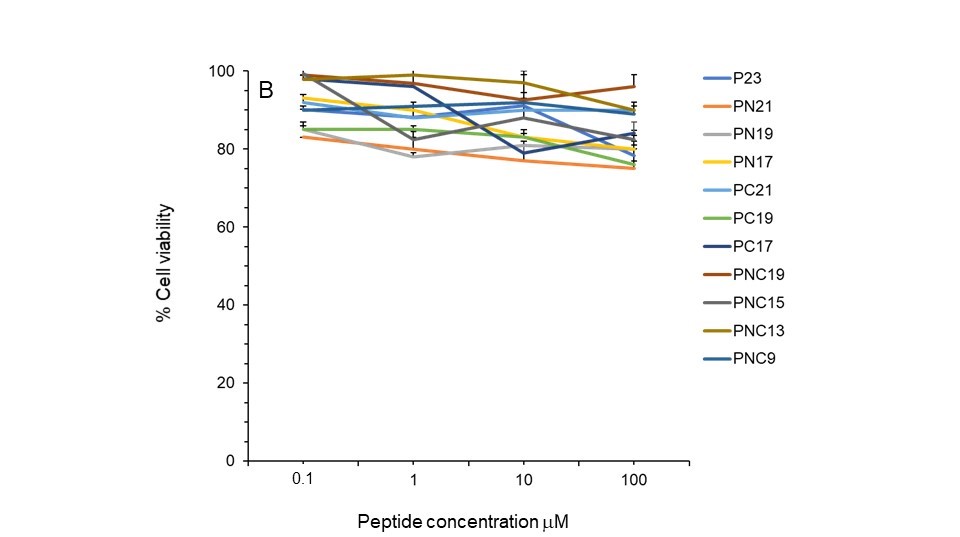


**Figure 26s.** Inhibitory activity and cytotoxicity of reduced size peptide P23 against SARS-CoV-2 Vero E6 cell infection. (A) SARS-CoV-2 infection of Vero E6 cells at MOI of 0.01 in presence of the indicated concentration of P23 and reduced size peptide was assayed with the viral plaque reduction assay. (B) Cell viability of Vero E6 cells in the same condition in absence of virus infection was assayed with MTT assay. The values shown are means + standard deviation of 3 independent experiments.


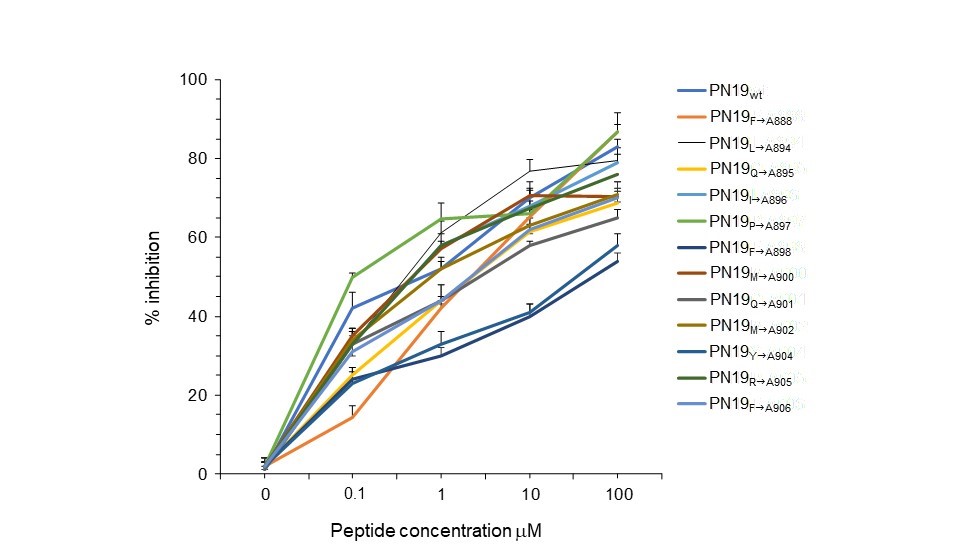


**Figure 27s.** Inhibitory activity of peptide PN19 mutated by alanine scan against SARS-CoV-2 Vero E6 cell infection. SARS-CoV-2 infection of Vero E6 cells at MOI of 0.01 in presence of the indicated concentration of PN19 mutated peptide was assayed with the viral plaque reduction assay. The values shown are means + standard deviation of 3 independent experiments.


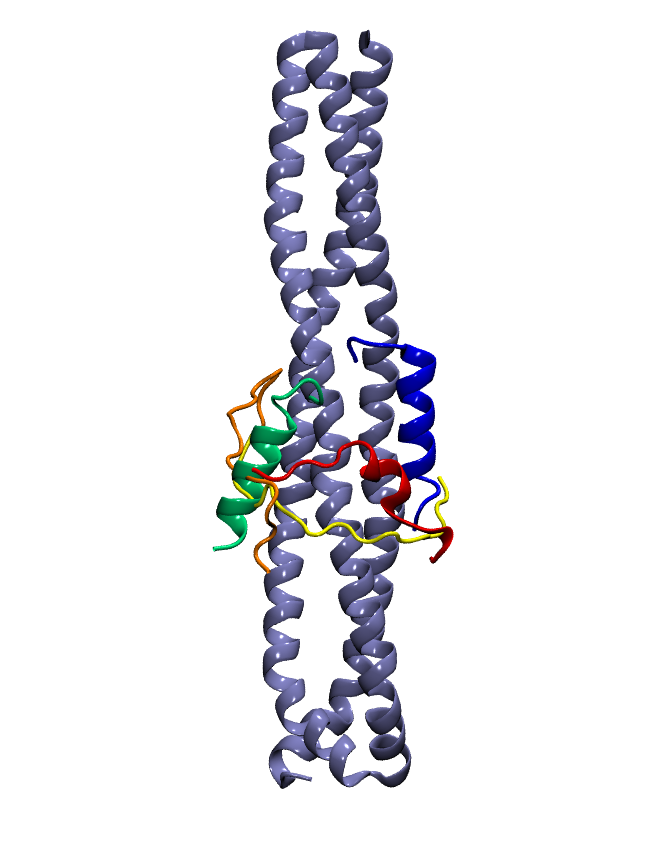


**Figure 28s.** PN19 binding pose with the highest docking score resulting from molecular docking on MPER models from ref. (Izvorski 2020). Only Model 1 of the receptor is reported in figure for clarity.

**Table 3s**. Predicted changes in binding free energy (∆∆Gbind) upon alanine mutation computed on MPER-PN19 complex with the highest docking score for each MPER model. Positive values mean that replacement by alanine is predicted to destabilize the complex.
